# Profiling the 3D interaction between germ cell tumors and microenvironmental cells at the transcriptome and secretome level

**DOI:** 10.1002/1878-0261.13282

**Published:** 2022-07-26

**Authors:** Margaretha A. Skowron, Katharina Eul, Alexa Stephan, Gillian F. Ludwig, Gamal A. Wakileh, Arthur Bister, Christian Söhngen, Katharina Raba, Patrick Petzsch, Gereon Poschmann, Edmund Osei Kuffour, Daniel Degrandi, Shafaqat Ali, Constanze Wiek, Helmut Hanenberg, Carsten Münk, Kai Stühler, Karl Köhrer, Elvira Mass, Daniel Nettersheim

**Affiliations:** ^1^ Department of Urology, Urological Research Laboratory, Translational UroOncology, Medical Faculty and University Hospital Düsseldorf Heinrich Heine University Düsseldorf Düsseldorf Germany; ^2^ Department of Urology and Paediatric Urology University Hospital Ulm Ulm Germany; ^3^ Department of Otorhinolaryngology and Head/Neck Surgery, Medical Faculty and University Hospital Düsseldorf Heinrich Heine University Düsseldorf Düsseldorf Germany; ^4^ Institute for Transplantation Diagnostics and Cell Therapeutics Medical Faculty and University Hospital Düsseldorf, Heinrich Heine University Düsseldorf Düsseldorf Germany; ^5^ Genomics and Transcriptomics Laboratory, Biological and Medical Research Centre (BMFZ) Medical Faculty and University Hospital Düsseldorf, Heinrich Heine University Düsseldorf Düsseldorf Germany; ^6^ Molecular Proteomics Laboratory, Biological and Medical Research Centre (BMFZ) Medical Faculty and University Hospital Düsseldorf, Heinrich Heine University Düsseldorf Düsseldorf Germany; ^7^ Clinic for Gastroenterology, Hepatology and Infectious Diseases Medical Faculty and University Hospital Düsseldorf, Heinrich Heine University Düsseldorf Düsseldorf Germany; ^8^ Institute of Medical Microbiology and Hospital Hygiene Medical Faculty and University Hospital Düsseldorf, Heinrich Heine University Düsseldorf Düsseldorf Germany; ^9^ Department of Pediatrics III University Children's Hospital Essen, University of Duisburg‐Essen Essen Germany; ^10^ Life and Medical Sciences (LIMES) Institute, Developmental Biology of the Immune System University of Bonn Bonn Germany

**Keywords:** cisplatin resistance, extracellular matrix, germ cell tumors, tumor microenvironment

## Abstract

The tumor microenvironment (TM), consisting of the extracellular matrix (ECM), fibroblasts, endothelial cells, and immune cells, might affect tumor invasiveness and the outcome of standard chemotherapy. This study investigated the cross talk between germ cell tumors (GCT) and surrounding TM cells (macrophages, T‐lymphocytes, endothelial cells, and fibroblasts) at the transcriptome and secretome level. Using high‐throughput approaches of three‐dimensional (3D) co‐cultured cellular aggregates, this study offers newly identified pathways to be studied with regard to sensitivity toward cisplatin‐based chemotherapy or tumor invasiveness as a consequence of the cross talk between tumor cells and TM components. Mass‐spectrometry‐based secretome analyses revealed that TM cells secreted factors involved in ECM organization, cell adhesion, angiogenesis, and regulation of insulin‐like growth factor (IGF) transport. To evaluate direct cell–cell contacts, green fluorescent protein (GFP)‐expressing GCT cells and mCherry‐expressing TM cells were co‐cultured in 3D. Afterward, cell populations were separated by flow cytometry and analyzed by RNA sequencing. Correlating the secretome with transcriptome data indicated molecular processes such as cell adhesion and components of the ECM being enriched in most cell populations. Re‐analyses of secretome data with regard to lysine‐ and proline‐hydroxylated peptides revealed a gain in proteins, such as collagens and fibronectin. Cultivation of GCT cells on collagen I/IV‐ or fibronectin‐coated plates significantly elevated adhesive and migratory capacity, while decreasing cisplatin sensitivity of GCT cells. Correspondingly, cisplatin sensitivity was significantly reduced in GCT cells under the influence of conditioned medium from fibroblasts and endothelial cells. This study sheds light on the cross talk between GCT cells and their circumjacent TM, which results in deposition of the ECM and eventually promotes a pro‐tumorigenic environment through enhanced migratory and adhesive capacity, as well as decreased cisplatin sensitivity. Hence, our observations indicate that targeting the ECM and its cellular components might be a novel therapeutic option in combination with cisplatin‐based chemotherapy for GCT patients.

## Graphical abstract

This study evaluated the interaction between germ cell tumors and their surrounding microenvironment. As such, the secretome has been analyzed to identify secreted factors, while transcriptome‐wide changes upon direct cell–cell interaction by means of 3D co‐cultures determined the deregulated gene sets. Subsequently, *in vitro* analyses were performed to validate the findings.

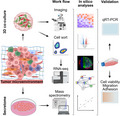

**Abbreviations**
3D: three‐dimensional
CAF: cancer‐associated fibroblast
CC: choriocarcinoma
CM: conditioned medium
DAPI: 4',6‐diamidino‐2‐phenylindole
DMEM: Dulbecco's Modified Eagle Medium
EC: embryonal carcinoma
ECM: extracellular matrix
EMT: epithelial‐to‐mesenchymal transition
FBS: fetal bovine serum
FC: fold change
FDR: false discovery rate
GCNIS: germ cell neoplasia *in situ*
GCT: germ cell tumors
GEO: gene expression omnibus
GFP: green fluorescent protein
GO: gene ontology
GSEA: gene set enrichment analyses
h: hours
IGF: Insulin‐like growth factor
LC‐MS: liquid chromatography‐mass spectrometry
LD_50_
: lethal dose
LPS: Lipopolysaccharide
min: minutes
ms: millisecond
PBMC: Peripheral blood mononuclear cell
PBS: phosphate‐buffered saline
PCA: principal component analysis
PMA: phorbol 12‐myristate‐13‐acetate
PMS: N‐methyl dibenzopyrazine methyl sulfate
qRT‐PCR: quantitative real‐time polymerase chain reaction
RPMI: Roswell Park Memorial Institute 1640 Medium
s: seconds
SE: seminoma
Seq: sequencing
STR: short tandem repeat
TAM: tumor‐associated macrophage
TE: teratoma
TM: tumor microenvironment
XTT: 2,3‐bis(2‐methoxy‐4‐nitro‐5‐sulfophenyl)‐5‐[(phenylamino)carbonyl]‐2H‐tetrazolium
YST: yolk‐sac tumor

## 1 Introduction

With more than 90 %, type II germ cell tumors (GCTs) represent the most malignant tumor entity among young men. Arising from germ cell neoplasia *in situ* (GCNIS), GCTs can be further distinguished between seminomas or non‐seminomas, such as embryonal carcinomas (EC) [1]. Representing malignant counterparts of embryonic stem cells, EC can further differentiate into teratomas (TE), yolk‐sac tumors (YST), or choriocarcinomas (CC) [2, 3]. The standard of care to treat type II GCTs comprises orchiectomy and subsequent chemo‐ or radiotherapy, eventually resulting in good response rates of about 90% [3]. However, the development of drug resistance precipitates poor prognosis and a short survival in about 10–15% of patients [4]. Cisplatin resistance has been noted in several tumor entities to be of multifactorial nature [5]. As a DNA intercalator resulting in intra‐ and interstrand crosslinks, cisplatin‐induced DNA adducts are often repaired by the nucleotide‐excision repair and translesion synthesis, or in case of double‐strand breaks by the homologous recombination [6, 7]. Eventually, cisplatin‐treated cells display a specific mutational signature (C > T and C > A) [5], resulting in diverse resistance mechanisms. However, another possible factor influencing the multifaceted development of cisplatin resistance is the surrounding tumor microenvironment, which is yet not fully understood. Stromal cells as well as the extracellular matrix (ECM) influence tumor cells by activating proliferative and anti‐apoptotic signaling pathways. Vice versa, the TM can be influenced by tumor cells as well [8, 9]. Also, the TM is known for its heterogeneous nature, eventually resulting in an antitumor immune microenvironment or immune suppressive microenvironment depending on contextual cues from the surrounding tissue [10].

Specifically, the testicular microenvironment has been ascribed an important role not only during developmental process of the testis, but also during the transition of misguided primordial germ cells to GCT cells [11]. The testis is structurally and functionally compartmentalized into seminiferous tubules and the interstitial component. While seminiferous tubules are lined by Sertoli cells and germ cells at a basal lamina, the interstitial space includes androgen‐producing Leydig cells, fibrocytes, and immune cells, such as tissue‐resident macrophages, mast cells, lymphocytes, natural killer‐, and dendritic cells [12, 13, 14]. While tumor‐infiltrating T‐lymphocytes have been attributed to the development of malignant extracranial GCT [15], PD‐L1^+^ tumor‐associated macrophages (TAM) have been detected rather in seminomas than nonseminomas [16]. Additionally, analysis of 22 types of tumor‐infiltrating immune cells revealed high infiltration of CD8+ T‐cells, macrophages, and dendritic cells in GCTs compared with normal samples [17]. In other tumor entities, such as pancreatic ductal adenocarcinoma, colorectal adenocarcinoma, or prostate cancer, highly differentiated and activated fibroblasts (cancer‐associated fibroblasts; CAF) were demonstrated to enhance tumor proliferation and metastatic capacity [18, 19, 20, 21], while inhibiting vascular‐like network formation [19]. As such, CAFs produce and secrete a number of soluble factors, which stimulate neighboring stromal cells to secrete further tumor growth‐supporting soluble factors, such as VEGF, HGF, TGFβ, IL6, CXCL12, and CCL2 [22]. In GCT cells, *miR‐125b* expression in tumor cells promoted a microenvironment enriched with TAM via increasing the production of tumor‐derived chemokines CSF1 and CX3CL1 for TAM recruitment [23]. Regarding the development of resistance, co‐culture of human stomach fibroblasts in conditioned media from gastric adenocarcinoma cell lines AGS or MGC‐803 led to enhanced IL‐8 secretion, eventually resulting in increased levels of NF‐κB and ABCB1 and consequently decreased cisplatin sensitivity in the tumor cells [24]. Hence, even though cisplatin‐based chemotherapy diminishes cancer cells, it possibly induces secretion of stroma‐derived factors to produce beneficial environments that promote resistance and increased tumor survival [25].

This study investigated the cross talk between GCT cells and their TM and its influence on the development of a pro‐tumorigenic environment resulting in diminished cisplatin sensitivity. We asked whether factors secreted by the TM might influence the cisplatin sensitivity of GCT cells and examined the interactions between GCT and TM cells using proteomics and transcriptomics *in vitro*. Hence, this study sheds light on the molecular events occurring during the interaction of GCT cells and their surrounding TM.

## 2 Materials and methods

### 2.1 Cell culture

The cultivation conditions and sources of GCT and noncancerous cell lines are described in Table S1 or have been published previously [26]. Short tandem repeat (STR) profiles are checked on a regular basis and are available upon request. Mycoplasma contamination is checked regularly by PCR strategy as described previously [27]. Differentiation and polarization of THP‐1 cells into macrophages have been performed as described by Genin et al. [28]. Briefly, incubation with 150 nm phorbol 12‐myristate 13‐acetate (PMA; Sigma‐Aldrich, Taufkirchen, Germany) resulted in the differentiation of THP‐1 cells into macrophages. Subsequent incubation with 20 ng·mL^−1^ of IFNg (Proteintech Germany GmbH, Planegg, Germany) and 10 pg·mL^−1^ of lipopolysaccharide (LPS; Sigma‐Aldrich) for 24 h or with 20 ng·mL^−1^ IL4 and 20 ng·mL^−1^ IL13 (both from R&D Systems, Wiesbaden, Germany) for 72 h initiated polarization into M^IFNg/LPS^ or M^IL4/IL13^ macrophages, respectively. Successful M^IL4/IL13^ polarization was evaluated routinely via qRT‐PCR of gene markers typical for a M2‐like signature (*CD14*, *CD36*, *CD68*, *CD163*, *CD206*, and *FN1*) and M1‐like signature (*IL10* and *CXCL10*) (Fig. S1A) or antibody staining with subsequent flow cytometry (CD14, CD36, and CD68) (Fig. S1B). The ethics committee of the Heinrich Heine University Düsseldorf (EC‐HHU‐D) raised no concerns about using analyzed cell lines for *in vitro* experiments and drug screening (ethics votes 2018–178 and 2019–412 to D. Nettersheim).

### 2.2 Processing of conditioned medium

Standard cell culture medium was conditioned by TM cells over 72 h. Depending on the proliferation rate, 3.75 × 10^6^ fibroblasts, 2.25 × 10^6^ JURKAT cells, 7.5 × 10^6^ M^IL4/IL13^ macrophages, and 1.125 × 10^6^ HUVEC cells were initially seeded in 15 cm cell culture dishes containing 17 mL medium. Afterward, conditioned medium (CM) was collected and pooled, sterile filtrated, and stored at −80 °C. Regarding harvesting of secretomes, GCT and TM cells were seeded in 15 cm cell culture dishes in standard cell culture medium. After 24 h, cells were washed five to seven times with 30 mL PBS before incubation in serum‐free medium for 24 h. Afterward, the medium was collected, centrifuged at 1000 ***g*** at 4 °C for 5 min, and filtered through a 0.2 μm Acrodisc syringe filter (VWR, Langenfeld, Germany) before being stored at −80 °C. As controls and for data normalization, the proteome of each cellular fraction was analyzed (each *n* = 3). Therefore, cells were harvested after being washed twice with 5 mL ice‐cold PBS. Cells were scraped into 1 mL of PBS, transferred into a 1.5‐mL tube, and centrifuged at 800 ***g*** at 4 °C. The supernatant was removed and the pellet stored at −80 °C. To evaluate the purity and quantity of the secretomes, a SDS/PAGE followed by a silver gel staining (Thermo Fisher Scientific, Schwerte, Germany) was performed after precipitation of 5 ml secretome by trichloroacetic acid.

### 2.3 Three‐dimensional cell co‐cultivation and cell sorting

As described previously [26], GFP^+^‐expressing GCT cell lines TCam‐2, 2102EP, NCCIT, JAR, JEG‐3, 1411H, and GCT72 and mCherry^+^‐expressing TM cell lines HUVEC, JURKAT, and THP‐1 have been established by transfecting 293T‐cells with the pczVSV‐G plasmid [29], pCD/NL‐BH∆1 plasmid (Addgene #41791 [30]), and puc2CL6EGIP [31] or pLV‐mCherry plasmid (Addgene #36084). Primary fibroblasts (MPAF and HVHF2) were stained with cell tracker dyes according to the manufacturer's protocol. Briefly, cells were stained with either 750 nm ‘CellTracker DeepRed Dye’ for flow cytometry or 2.5 μm ‘CellTracker CM‐DiI Dye’ for microscopy (both Thermo Fisher Scientific) in prewarmed serum‐free medium or PBS, respectively, for 20 min at 37 °C. Confirmation of staining has been shown previously [26]. The three‐dimensional co‐cultivation of GCT and TM cells (3 × 10^3^ cells per 40‐μL drop) has been performed as described previously [26, 32].

### 2.4 Measurement of cell viability

XTT viability assays were performed as described previously [26]. Briefly, 1–3 × 10^3^ cells were plated onto 96‐well plates before treatment with cis‐diamminedichloroplatinum‐II (Cisplatin; Accord Healthcare, London, UK) for up to 96 h. Regarding the effect of conditioned media on cisplatin sensitivity, GCT cells were pretreated with CM from TM cells for 24 h before cisplatin treatment. Every day viability was screened by adding 50 μL 2,3‐bis(2‐methoxy‐4‐nitro‐5‐sulfophenyl)‐5‐[(phenylamino)carbonyl]‐2H‐tetrazolium (XTT; 1 mg· mL^−1^;neoLab Migge GmbH, Heidelberg, Germany) and 0.5 μL N‐methyl dibenzopyrazine methyl sulfate (PMS; 1.25 mm; Sigma‐Aldrich) and measuring absorbance 4 h later in a UV/VIS spectrometer (450 nm vs. 650 nm, iMark Microplate Absorbance Reader, BioRad, Feldkirchen, Germany). Each time point/concentration was measured in quadruplicates. LD_50_ doses were calculated using graphpad prism software version 8 (GraphPad Software, San Diego, CA, USA).

### 2.5 Migration and adhesion assay

To evaluate the influence of ECM components, culture plates were coated with collagen I from calf skin, collagen IV from human placenta, hyaluronic acid, sodium salt from *Streptococcus equi*, fibronectin from human plasma, or synthetic laminin peptide (all from Sigma‐Aldrich). For cell migration and cell adhesion assay, cell culture plates were coated with collagen I (66 μg·mL^−1^; 10 μg·cm^−2^), collagen IV (100 μg·mL^−1^; 15 μg·cm^−2^), hyaluronic acid (1 mg·mL^−1^; 150 μg·cm^−2^), fibronectin (20 μg·mL^−1^; 3 μg·cm^−2^), or laminin (150 μg·mL^−1^; 22.5 μg·cm^−2^) overnight at 37 °C before being washed once with PBS and dried for 30 min at 37 °C. Adhesion assay has been performed as described previously [26]. For migration assays, two‐well culture inserts (ibidi GmbH, Gräfelfing, Germany) were placed in 24‐well plates, filled with cell suspension (3.5 × 10^4^–5.25 × 10^4^ cells per insert well) in standard culture medium, and incubated overnight at 37 °C. Subsequently, a mark was drawn on the back of the wells and the culture medium was replaced with medium containing 10 μg·mL^−1^ mitomycin C (Sigma‐Aldrich) for 2 h. Afterward, the medium and the culture inserts were carefully removed and the 24‐well was filled with standard culture medium. The ‘image j’ plugin described by Suarez‐Arnedo et al. has been utilized for the quantification of *in vitro* migration assays [33].

### 2.6 Quantitative RT‐PCR and RNA sequencing

RNA was isolated using the RNAeasy Mini Kit (Qiagen, Hilden, Germany) according to the manufacturer's protocol. *In vitro* transcription or RNA and qRT‐PCR was performed as described previously [26]. Gene expression was determined on the 384‐well C1000 cycler (BioRad) with oligonucleotides given in Table S2. *GAPDH* and *ACTB* were used as housekeeping genes and for data normalization. RNA samples used for transcriptome analyses were assessed as described previously [26, 27, 34]. Only RNA with an integrity number of > 8.5 was analyzed. RNA‐sequencing data are freely available via GEO. The data are available via the NCBI Gene Expression Omnibus (GEO) database repository (GSE195794; https://www.ncbi.nlm.nih.gov/geo/).

### 2.7 Flow cytometry and confocal microscopy

To evaluate successful THP‐1‐M^IL4/IL13^ differentiation [28], the number of CD36 positive cells was evaluated using CD14 (REA599) and CD36 (REA760) antibodies conjugated to APC diluted 1 : 100 as described previously using the cell surface staining protocol [26]. For CD68‐APC (REA886) staining, cells were fixed with 4% formaldehyde for 10 min and permeabilized for 5 min in 0.1% Tween‐20 in PBS before incubation with 1 : 100 diluted antibody for 30 min. Measurement was performed utilizing a ‘MACSQuant’ flow cytometer and ‘Flowlogic’ software (all from Miltenyi Biotec, Bergisch Gladbach, Germany).

For cell sorting, a ratio of GCT cell lines and TM cells of 30 : 70 was used. Depending on the co‐culture, 3 × 10^3^–6.5 × 10^3^ hanging drops were seeded. Cell sorting of 7–30 × 10^6^ cells was performed on a MoFlo XDP (Beckman Coulter, Krefeld, Germany). The protocol used for flow cytometry consisted of an initial discrimination of GFP^+^ (GCT) and mCherry^+^ or DeepRed^+^ (TM) cells. The width and height of the side‐scatter signals (SSC‐width, SSC‐height) was used to isolate single events from cellular aggregates and debris. Single GFP^+^/mCherry^−^ and GFP^−^ / mCherry^+^ or GFP^+^ / DeepRed^−^ and GFP^−^ / DeepRed^+^ cells were sorted into individual 15‐mL falcon tubes to be directly processed for RNA extraction. qRT‐PCR has been performed to test for purity of distinct GCT or TM cell populations [26]. For imaging, hanging drop co‐cultures were incubated at a ratio of 70 : 30 (GCT cell: TM cells) for up to 72 h. The resulting cell aggregates were fixed with 50 μL 3.7% paraformaldehyde for 1.5 h at room temperature and then transferred to a 96‐well plate. Counterstaining with DAPI was performed to visualize nuclei. Cell aggregates were imaged on uncoated black 96‐well μ‐plates (ibidi GmbH) using Plan‐Apochromat 10×/0.45 M27 and Plan‐Apochromat 40×/0.95 Korr M27 objective on the Zeiss LSM 710 (Argon 405, 488, and 543 lasers; Carl Zeiss AG, Oberkochen, Germany). In addition, z stacks were recorded for 3D projections and proceeded using the zen software (Carl Zeiss AG). For further image processing, fiji‐2 software was used [35].

### 2.8 Mass spectrometry: LC–MS analysis

For the identification of secreted proteins using liquid chromatography (LC) coupled with MS, CM of cells and cell lysates were prepared for secretome analysis as described in Grube et al. [36]. Briefly, proteins were precipitated by trifluoroacetic acid (conditioned medium), lysates prepared in urea/thiourea containing buffer (30 mm tris(hydroxymethyl)aminomethane, 2 m thiourea, 7 m urea and 4% (w/v) 3‐[(3‐cholamidopropyl)dimethylammonio]‐1‐propanesulfonate, in H_2_O, pH 8.5) and proteins shortly separated in polyacrylamide gels, reduced with dithiothreitol, alkylated with iodoacetamide and subjected to in‐gel digestion with trypsin. Resulting peptides were separated using an Ultimate 3000 rapid separation liquid chromatography system (Thermo Fisher Scientific) on C18 material using a 2 h gradient essentially as described previously [36]. Subsequently, peptides were injected via a nano‐electrospray interface into an Orbitrap Fusion Lumos mass spectrometer (Thermo Fisher Scientific). The mass spectrometer was operated in data‐dependent positive mode. After recording precursor‐spectra in the orbitrap (profile mode, resolution: 120 000, scan range: *m*/*z* 200–2000, maximum injection time: 60 ms, advanced gain control target: 250 000), twofold–sevenfold charged precursors were isolated (*m*/*z* 1.6 isolation window), fragmented using higher‐energy collisional dissociation (collision energy 35%), and analyzed in the linear ion trap of the instrument (centroid mode, scan rate: ‘rapid’, maximum injection time: 50 ms, advanced gain control target: 100 00). After 2 s, a new cycle started and already fragment precursors were excluded from further fragmentation for the next 60 s. Protein identification and quantification was carried out with MaxQuant (version 1.6.12.0, Max Planck Institute for Biochemistry, Planegg, Germany) using standard parameters if not stated otherwise. Searches were carried out on the basis of 74 811 sequences from the homo sapiens proteome dataset (UP000005640, UniProt KB, downloaded on 27th March 2020). Methionine oxidation and N‐terminal acetylation were considered as variable and carmbamidomethylation at cysteines considered as fixed modifications. The ‘match between runs’ function was enabled as well as iBAQ and label‐free quantification. Peptides and proteins were identified at a false discovery rate of 1 % and only proteins identified with at least two different peptides and three valid intensity values in at least one group (conditioned medium or cell lysate) considered for further analysis. Protein groups were annotated with information about signal peptides, transmembrane domains, and KDEL sequences with data downloaded from the UniProt KB on 27th March 2020. Unconventional protein secretion was predicted with OutCyte [37]. The mass spectrometry proteomics data have been deposited to the ‘ProteomeXchange Consortium’ via the ‘PRIDE’ [38] partner repository with the dataset identifier PXD031329. For further analyses, we focused on all proteins significantly enriched in the supernatant compared with the cellular fraction and excluded all proteins classified as potential contaminants from further analyses. Subsequently, another MaxQuant search was carried out including identification and quantification of lysine and proline‐hydroxylated peptides (variable modification) with iBAQ quantification enabled. Secreted proteins were determined by OutCyte and extracellular matrix and extracellular vesicular proteins annotated by information from UniProt knowledge base (data downloaded on January 18, 2022). Proteins were filtered for proteins predicted to be secreted by OutCyte and showed at least 10 valid values over all cell lines. The top 150 mean protein intensities were used to build voronoi treemaps for each cell line.

### 2.9 Cytokine array

The C‐Series Human Cytokine Antibody Array 2000 Kit (RayBiotech/Hölzel Diagnostika Handels GmbH, Cologne, Germany) has been performed in duplicates according to the manufacturer's protocol to evaluate secreted factors in the supernatant from GCT cell lines and TM cells. Analysis of spots (*n* = 4) has been evaluated using the ‘Protein Array Analyzer’‐Plugin [39] for ‘image j' (https://imagej.nih.gov/ij/) [40]. Significant signals were set to a threshold >8500 arbitrary gray value.

### 2.10 Online analyses tools

The STRING algorithm was used to predict protein–protein‐interaction by confidence (https://string‐db.org) [41]. Prediction of molecular functions of deregulated genes/proteins found in RNA‐seq or mass spectrometry was analyzed using ‘DAVID Functional Annotation Tool’ based on ‘GOTERM_BP_DIRECT’, ‘UP_KEYWORDS’ and ‘KEGG_PATHWAY’ (https://david.ncifcrf.gov) [42] and was visualized using the online platform ‘ImageGP’ (http://www.ehbio.com/ImageGP/) to generate dot plots [43]. The ‘pandas’, ‘seaborn’, and ‘matplotlib’ libraries were used in ‘Python’ for statistical data visualization of RNA‐seq data via volcano and box plots [44, 45, 46, 47]. ‘PCAGO’ (https://pcago.bioinf.uni‐jena.de) was used to perform 3D principal component analyses (PCA) of RNA‐seq and mass spectrometry data [48]. A Pearson correlation matrix online software (http://www.sthda.com/english/rsthda/correlation‐matrix.php) was used to generate a correlogram from secretome data [49]. Voronoi treemaps have been visualized using the ‘shinytreemaps' application (https://m‐jahn.shinyapps.io/ShinyTreemaps/) [50]. The browser‐based tool ‘beavr’ was utilized to create hierarchical sample clustering and gene set enrichment analyses (GSEA) (FDR correction 10%), Shrinkage estimator: Normal (adaptive normal distribution, genes below 10 reads were excluded from analysis) of RNA‐seq data [51]. Venny 2.1 was utilized to display venn diagrams (https://bioinfogp.cnb.csic.es/tools/venny/index.html) [52]. Graphical illustrations have been created with BioRender.com.

### 2.11 Statistical analyses

To analyze differences between groups, two‐tailed Student's *t*‐tests have been performed after confirming equality of two variances by means of *F*‐tests. Statistically significant differences are highlighted by asterisk (*P < 0.05, **P < 0.005, ***P < 0.0005). Standard deviations are shown as error bars.

## 3 Results

**Fig. 1:**
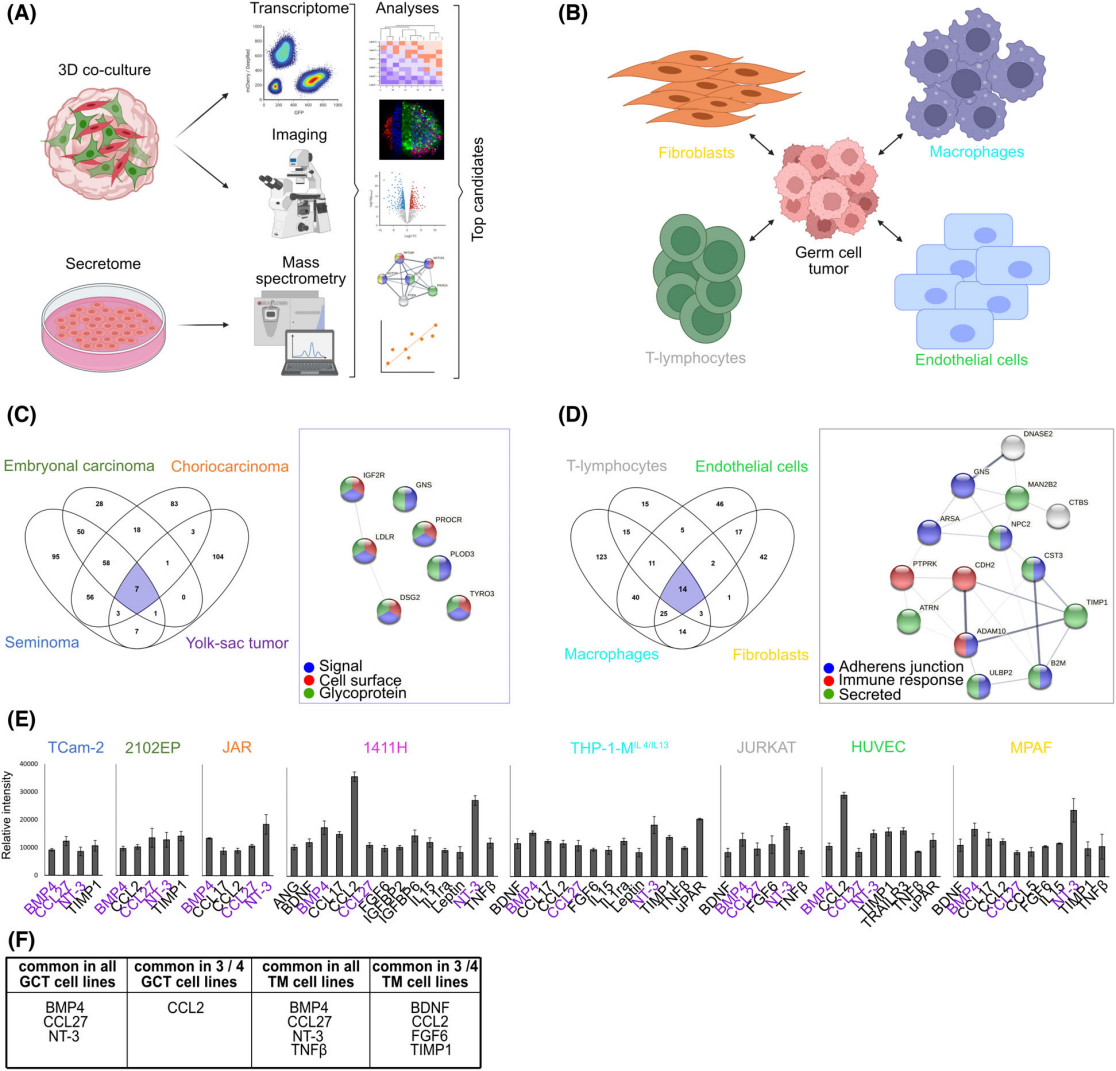
Analysis of factors secreted by either GCT or microenvironmental cells. Graphical illustration of the (A) methods and (B) cell lines used in this study (created with BioRender.com). (C) Venn diagram of factors commonly secreted from GCT cells (*n* = 3). STRING interaction analysis of secreted factors commonly found in all four GCT cells. (D) Venn diagram of factors commonly secreted from TM cells (*n* = 3). STRING interaction analysis of secreted factors commonly found in all four TM cells. (E) Most prominent secreted proteins from GCT cell lines (TCam‐2, 2102EP, JAR, 1411H) and TM cells (THP‐1‐M^IL4/IL13^, JURKAT, HUVEC, and MPAF). Commonly secreted proteins found in GCT cell lines, TM cells, or in at least three out of four GCT cell lines or TM components as measured by cytokine arrays (*n* = 4) are highlighted with purple protein names and have been summarized in (F). Standard deviations are shown as error bars. GCT, germ cell tumor; TM, tumor microenvironment.

In order to investigate the interaction between GCT cells and TM cells, this study utilized proteomics to identify secreted factors by mass spectrometry (MS), as well as transcriptome data of GFP‐transduced GCT cells and mCherry‐transduced TM cells being co‐cultured in a 3D hanging drop model before separations of both populations by flow cytometry (Fig. 1A). Further, confocal microscopy visualized the morphological changes during the development of cell aggregates upon 3D co‐culture (Fig. 1A). In total, six germ cell tumor cell lines (seminoma: TCam‐2; EC: 2102EP, NCCIT; CC: JAR, JEG‐3; YST: GCT72; EC‐YST‐intermediate: 1411H) and five TM cell lines (fibroblasts: MPAF, HVHF2; M^IL4/IL13^ macrophages: THP‐1 polarized into M^IL4/IL13^ macrophages; T‐lymphocytes: JURKAT; endothelial cells: HUVEC) have been included in this study (Fig. 1B).

### 3.1 Direct and indirect cross talk between GCT cells and microenvironmental components organizes extracellular matrix

To evaluate the factors secreted by either GCT or TM cells, supernatants of GCT and TM cells were collected and the secretome analyzed by MS (each *n* = 3) (Fig. 1A; Table S3A–L). At the time point of supernatant isolation, all cells were highly viable and isolated secretomes were quality controlled by silver stainings prior to MS (Fig. S1C,D). Comparing the secretomes by a hierarchical clustering heatmap demonstrated that GCT cells clustered apart from the microenvironmental cells (Fig. S1E). A PCA showed that within the GCT cells, JAR and JEG‐3 (CC) grouped together, and TCam‐2 (seminoma), 2102EP, NCCIT (EC), and 1411H (EC‐YST) clustered together, while GCT72 (YST) presented a more unique secretome profile (Fig. S1F). Regarding the secretome profile of TM cells, only the fibroblast cells HVHF2 and MPAF grouped closely, but in general the microenvironmental components clustered clearly apart from each other (Fig. S1G). Next, a correlation matrix has been performed to investigate the dependence between the various secretome profiles (Fig. S1H). We noticed a resemblance between JURKAT and HUVEC cells with seminoma, CC, and EC cells (Fig. S1H; Table S3A–L). We identified all proteins secreted by seminoma (TCam‐2), EC (2102EP and NCCIT), choriocarcinoma (JAR and JEG‐3), and yolk‐sac tumor cell lines (GCT72) as well as fibroblasts (MPAF and HVHF2), endothelial cells (HUVEC), M^IL4/IL13^ macrophages (THP‐1‐ M^IL4/IL13^) and T‐lymphocytes (JURKAT) (Figs S1F–H, S2 and S3; Table S3M–U). Additionally, we identified all proteins commonly secreted by GCT or TM cells (Fig. 1C,D; Table S3R,S). Furthermore, we identified all molecules secreted exclusively by GCT cells (not in any TM cell) or TM cells (not in any GCT cell line) (Table S3T,U). As shown by gene ontology (GO) analyses and predicted by the STRING algorithm, proteins secreted from GCT or TM cells were involved in ‘cell–cell adhesion’ and ‘extracellular matrix organization’ (Figs S2 and S3; Table S3R–U). Additionally, factors involved in signaling pathways, such as ‘JAK–STAT’, ‘tyrosine kinase activity’, ‘integrin binding’, or ‘IGF‐receptor’ were specifically secreted from seminoma cells, choriocarcinoma cells, endothelial cells, and fibroblasts, respectively (Figs S2A,C and S3B,D). Moreover, M^IL4/IL13^ macrophages secreted factors relevant for regulating the immune response, leukocyte differentiation, and regulation of T‐cell activation (Fig. S3A). TYRO3, which has been recently identified as a potential therapeutic target in urothelial carcinoma [53], has been identified as one of the seven commonly secreted proteins from GCT cell lines (Fig. 1C). Factors commonly ‘secreted’ from TM cells could be linked to ‘immune response’ and ‘adherence junction’ (Fig. 1D). In line with the role of polarized M^IL4/IL13^ macrophages in providing an anti‐inflammatory and tumor‐promoting microenvironment, we propose that the proteins in this postulated network are involved in these processes (Fig. S3A). Of note, in JURKAT T‐lymphocytes, we detected only 13 exclusively secreted proteins in total (vs. all GCT cells) and only two of these proteins (CD6 and CD28) were predicted to interact with each other (Fig. S3C; Table S3U). Thus, the influence of the T‐lymphocyte secretome on GCT cells seems to be rather weak.

To extend these findings and to identify the most prominent signaling molecules, cytokine arrays of the secretomes of GCT cell lines (TCam‐2, 2102EP, JAR, and 1411H) and TM cells (THP‐1‐M^IL4/IL13^, JURKAT, HUVEC, and MPAF) have been performed (threshold: > 8500 arbitrary gray value) (Fig. 1E; Fig. S4; Table S3V, labeled in dark red). BMP4, CCL27, and NT‐3 were secreted commonly by all GCT cell lines and TM cells (Fig. 1F). Moreover, TM components commonly secreted TNFβ. Additionally, CCL2 was commonly secreted in three out of four GCT cell lines, while BDNF, CCL2, FGF6, and TIMP1 were commonly secreted in three out of four TM cells (Fig. 1F; Fig. S4; Table S3V).

Hence, the MS‐based secretome analyses as well as cytokine arrays identified specific growth factors involved in signaling cascades that eventually could influence the respective neighboring cell population. Thus, the influence of direct cell–cell connection between GCT cells and TM components was evaluated on transcriptome level of the respective cell population upon 3D co‐culture.

Therefore, GCT cells were co‐cultured with each TM cell type for 72 h using the 3D hanging drop cell culture technique. Using a 3D approach, the cellular aggregates can mimic the natural structure of the *in vivo* scenario, thereby enabling also further analyses regarding cell–cell interactions [54]. In comparison with other 3D models using liquid overlays or scaffold matrices, the hanging drop model uses solely gravitational forces to form cell aggregates, thereby eliminating additional factors provoked by synthetic alternatives, such as collagen‐containing hydrogels [55].

In 3D mono‐cultures, the EC and seminoma cell lines (2102EP, NCCIT and TCam‐2) formed roundish spheres, while the other tumor subtypes, as well as TM cells formed more irregularly shaped aggregates (Fig. S5A,B). Next, we illustrated the cellular arrangements of each cell type in co‐culture by confocal microscopy (GCT: GFP^+^; TM: mCherry^+^; MPAF/HVHF2: CM‐Dil stained) (Fig. S5C). We observed that fibroblasts (MPAF and HVHF2), endothelial cells (HUVEC), and T‐lymphocytes (JURKAT) were mostly equally distributed throughout the aggregates, while M^IL4/IL13^ macrophages were found in clusters on the surface of the aggregates (Fig. S5C). Though, suspecting a rather stochastic distribution of cells, Chen and Zou observed using a mathematical multiscale method in 3D cell aggregates that differential adhesive forces among heterotypical cells resulted in various aggregation patterns [56]. Beyond that, a possible reason for the formation of clusters surrounding the aggregates could be the development of a hypoxic core in the cell aggregate, which has been also reported in 3D cultured colorectal as well as head and neck cancer cells, and human adipose tissue‐derived mesenchymal stem cells [57, 58, 59]. Since the hypoxia inducible factor HIF‐1α is known to activate signaling pathways regulating epithelial‐to‐mesenchymal‐transition (EMT), as well as ECM remodeling [60, 61], motility of GCT cells and TM components within a cellular aggregate could be rather plastic due to enhanced ECM re‐organization within both populations.

Transcriptome‐wide changes in each GCT/TM cell population were evaluated after 3D co‐cultivation and subsequent separation by flow cytometry (Fig. S6). The purity of flow cytometry‐sorted populations was validated by qRT‐PCR using specific marker genes (GCT: *EpCAM* / *CD326*; TM cells: *CD44*, fibroblasts: *DCN*, endothelial cells: *PECAM1/CD31*, lymphocytes: *CD6*, macrophages: *CD36*) [26] (Fig. S7A,B).

PCA and hierarchical cluster analyses of RNA‐sequencing (RNA‐seq) data revealed that sorted GCT cell populations clustered to their corresponding mono‐3D‐cultures, but clearly clustered apart from each other (220–7032 deregulated annotated genes, *P*‐value false discovery rate (FDR) < 0.05, fold change (FC) log_2_ 1.5) (Fig. S7C,D,G). A PCA and hierarchical clustering of TM cells upon co‐culture compared with their mono‐culture indicated a distinct separation of all four TM components (137–2930 deregulated annotated genes, *P*‐value FDR < 0.05, FC log_2_ 1.5) (Fig. S7E,F,H).

**Fig. 2:**
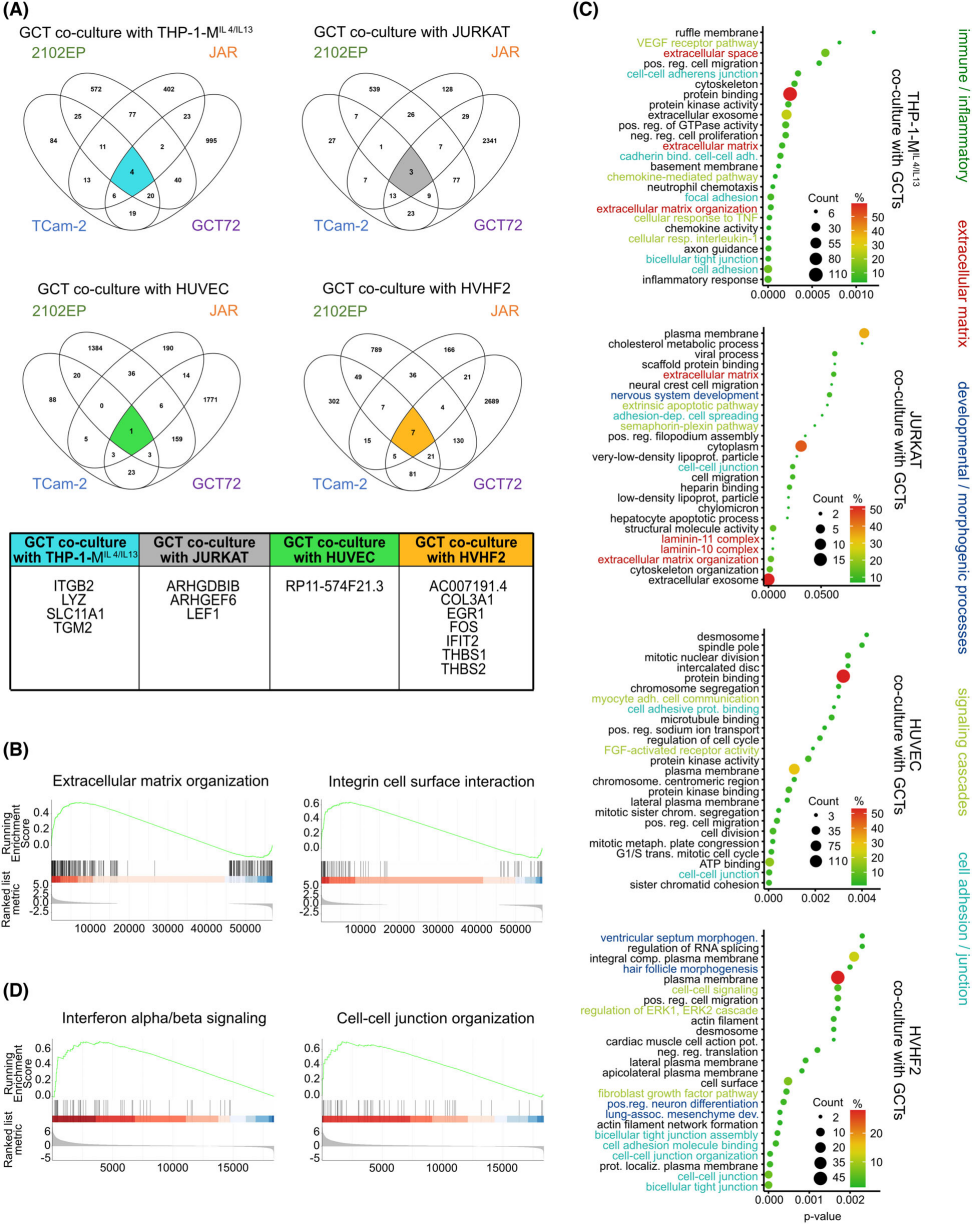
Transcriptome analysis after 3D co‐culture of GCT cells with TM cells and vice versa. (A) Venn diagrams of commonly deregulated genes found in TCam‐2, 2102EP, JAR, and GCT72 cells upon 3D co‐culture with TM cells. (B) GSEA of GCT cell lines (*n* = 4: TCam‐2, 2102EP, JAR, and GCT72) co‐cultured with TM cells HUVEC, JURKAT, THP‐1‐M^IL4/IL13^, and HVHF2 compared with mono‐cultured GCT cell lines indicate ‘extracellular matrix organization’ and ‘integrin cell surface interaction’ being among the top significantly deregulated gene sets. (C) Gene enrichment analyses of upregulated genes found in THP‐1‐M^IL4/IL13^, HUVEC, JURKAT, or HVHF2 cells co‐cultured with GCT cells (*n* = 4). (D) GSEA of TM cells (*n* = 4: HUVEC, JURKAT, THP‐1‐M^IL4/IL13^, and HVHF2) co‐cultured with GCT cell lines cells TCam‐2, 2102EP, JAR, and GCT72 compared with mono‐cultured TM cells indicate ‘interferon alpha/beta signaling’ and, ‘cell‐cell junction organization’ being among the top significantly deregulated gene sets. GCT, germ cell tumor; GSEA, gene set enrichment analysis; TM, tumor microenvironment.

Next, we performed GO enrichment analyses of all genes upregulated in TCam‐2, 2102EP, JAR, and GCT72 after co‐culture with THP‐1‐M^IL4/IL13^, JURKAT, HUVEC, or HVHF2 compared with the related mono‐culture to identify alterations in underlying biological processes or functions. Most of the top 25 enriched GO terms indicated that the upregulated genes were mainly involved in immune/inflammatory response, extracellular matrix, developmental/morphogenic processes, or transcriptional regulation (Fig. 2A).

A combined gene set enrichment analyses (GSEA) of all GCT cell lines (TCam‐2, 2102EP, JAR, and GCT72) co‐cultured with TM cells HUVEC, JURKAT, THP‐1‐M^IL4/IL13^, and HVHF2 compared with mono‐cultures indicated that processes like ‘extracellular matrix organization’ and ‘integrin cell surface interaction’ were among the top significantly deregulated gene sets (Fig. 2B).

Analyzing the enriched gene sets in each TM component after co‐cultivation with the GCT cells demonstrated that genes involved in ‘extracellular matrix’, ‘developmental/morphogenic processes’, ‘cell–cell communication’ (e. g. via cytokines), ‘immune/inflammatory response’, ‘signaling cascades’, and ‘cell adhesion/junction’ (Fig. 2C). Potential interactions between genes enriched in TM cells upon co‐culture with GCT cells indicated that transcripts involved in ‘cell adhesion/junction’ were commonly upregulated (Fig. S8). GSEA of all TM cells co‐cultured with GCT cell lines cells compared with mono‐cultures indicated ‘interferon alpha/beta signaling’ and ‘cell‐cell junction organization’ as the top enriched gene sets (Fig. 2D).

**Fig. 3:**
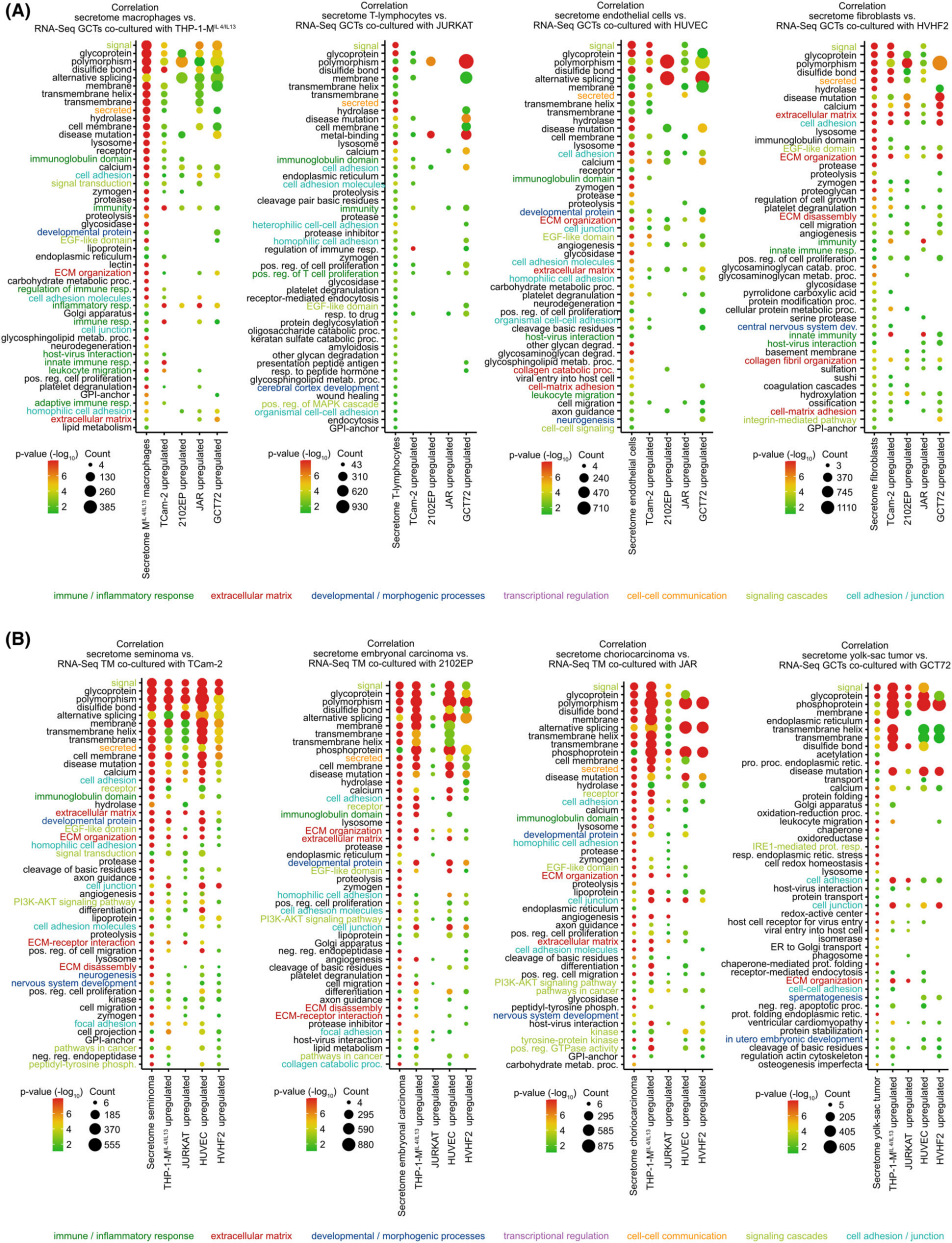
Correlation of secretome data to changes on transcriptome level. (A) Secretome enrichment analyses from TM cells (M^IL4/IL13^ macrophages: THP‐1‐M^IL4/IL13^; T‐lymphocytes: JURKAT; endothelial cells: HUVEC; fibroblasts: HVHF2, MPAF) and the correlation to biological processes being upregulated in GCT cells (TCam‐2, 2102EP, JAR, GCT72) upon 3D co‐culture with corresponding TM cells (HUVEC, JURKAT, THP‐1‐M^IL4/IL13^, HVHF2). (B) Secretome enrichment analyses from GCT cells (seminoma: TCam‐2; embryonal carcinoma: 2102EP, NCCIT; choriocarcinoma: JAR, JEG‐3, yolk‐sac tumor: GCT72) and the correlation to biological processes being upregulated in TM cells (HUVEC, JURKAT, THP‐1‐M^IL4/IL13^, and HVHF2) upon 3D co‐culture with corresponding TM cells (TCam‐2, 2102EP, JAR, and GCT72). GCT, germ cell tumor; TM, tumor microenvironment.

Next, we correlated the secretome to transcriptome data. GO enrichment analyses of the secreted factors from TM cells were correlated with the GO enrichment analyses of genes deregulated in GCT cells after co‐culture with the respective TM cell line (Fig. 3A) and vice versa (Fig. 3B). By this, we aimed at identifying factors secreted from TM cells, which were able to induce corresponding signaling cascades in GCT cells (and vice versa). We demonstrated that in both, GCT and TM cells, alterations in the secretome and transcriptome could be linked to the GO terms ‘cell adhesion’ and ‘extracellular matrix’ (except for JURKAT) (Fig. 3A,B). Of note, secreted factors from M^IL4/IL13^ macrophages as well as JURKAT lymphocytes could be linked to an ‘immune/inflammatory response’; consequently, similar gene sets were enriched in GCT cells upon co‐culture with THP‐1‐M^IL4/IL13^ or JURKAT cells (Fig. 3A).

Thus, summarizing our observations to this point, we concluded that GCT cells as well as TM components cells mainly secreted growth factors that resulted in transcriptome‐wide changes provoking modifications in the organization of the ECM as well as cell adhesion.

### 3.2 The extracellular matrix is involved in GCT invasiveness

**Fig. 4:**
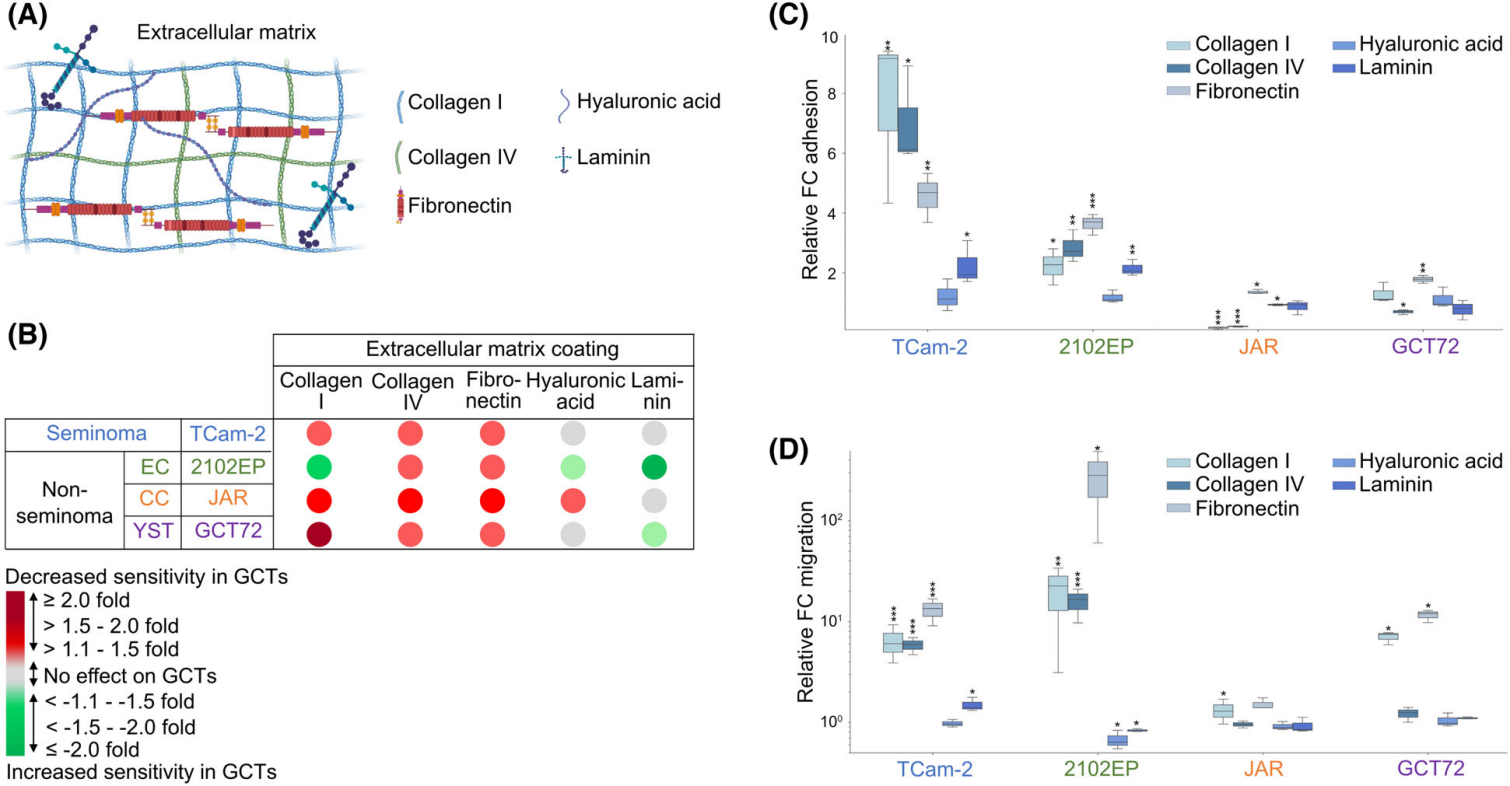
The ECM reduces cisplatin sensitivity and enhances migration in GCT cells. (A) Graphical illustration of used coatings representing different components of the ECM (created with BioRender.com). (B) Relative cell viability after 72 h of cisplatin treatment in GCT cells (TCam‐2, 2102EP, NCCIT, JAR, GCT72) being seeded on ECM‐coated culture plates (collagen I, collagen IV, fibronectin, hyaluronic acid, and laminin). Indicated as fold change compared with respective uncoated control (*n* = 4). (C) Adhesion (*n* = 3) and (D) migration assay (*n* = 3) of GCT cells (TCam‐2, 2102EP, NCCIT, JAR, and GCT72) seeded on ECM‐coated culture plates (collagen I, collagen IV, fibronectin, hyaluronic acid, and laminin). Two‐tailed *t*‐tests were performed to test for significance; *P < 0.05, **P < 0.005, and ***P < 0.0005. Standard deviations are shown as error bars. ECM, extracellular matrix; GCT, germ cell tumor; TM, tumor microenvironment.

So far, we have identified the growth factors being secreted from GCT cells and TM components that could result in the transcription of genes relevant for the ECM organization. Our observations led us to conclude that the ECM could have a pivotal role regarding the invasiveness of GCT. Specifically, the organization and degradation of the ECM has been attributed to play a major role regarding cisplatin sensitivity [62]. Moreover, enhanced ECM structures were reported to be crucially involved during tumor invasiveness due to enhanced migratory capacity while metastasizing and cell adhesion during the process of seeding [63]. Thus, we re‐analyzed our previously described secretome data with the emphasis on ECM components and included lysine and proline‐hydroxylated peptides (Table S3W,X). Globally, compared with the first MS secretome analyses, the gain in identified proteins and peptides was marginable (4 more identified proteins, additional 1.6% peptides, 0.3% unique peptides, and 0.25% quantitative values). The majority of higher abundant proteins was found to be hydroxylated at lysine or proline residues, though this was especially the case for ECM proteins, such as collagens and fibronectin (Table S3W,X; Fig. S9). Of note, with regard to ligands to receptor‐mediated signaling, collagens and fibronectin have been reported to bind to integrin receptors eventually stimulating adhesion and migration of tumor cells [64, 65]. As such, specifically *ITGA4* expression was elevated upon co‐culture of TCam‐2 cells with most TM components, while *ITGA1/2B/3/8* and *ITGB4/7/L1* were often upregulated in GCT72 cells after co‐culture with TM components (Table S3Y). Thus, we hypothesized that the elevated expression of integrins could be justified by surrounding ECM components resulting from the interaction between both cell populations. We next investigated the role of the ECM in GCT cells and elucidated its involvement in cisplatin sensitivity, cell migration, and cell adhesion. Collagen I, collagen IV, fibronectin, hyaluronic acid, and laminin were chosen for further investigations as being the most frequently occurring ECM components (Fig. 4A). Cultivation of GCT cell lines (TCam‐2, 2102EP, JAR, and GCT72) on cell culture plates precoated with collagen I, collagen IV, or fibronectin decreased cisplatin sensitivity after 72 h compared with uncoated controls (Fig. 4B). However, this effect was not observed in GCT cells seeded on plates precoated with hyaluronic acid or laminin (Fig. 4B). Subsequently, we evaluated the effect of these ECM components on cell adhesion and migration. Similar to our previous observations, particularly collagen I, collagen IV, and fibronectin enhanced cell adhesion (Fig. 4C), as well as the migratory capacity (Fig. 4D) significantly in most GCT cell lines compared with their respective uncoated controls. Again, hyaluronic acid or laminin did not have an influence on GCT adhesion or migration (Fig. 4C,D).

Summarizing, based on the re‐analysis of secretome data, we could identify collagen I, collagen IV, and fibronectin as ECM components affecting cisplatin sensitivity, while modulating migratory and adhesive capacity of GCT cells.

### 3.3 Secreted factors from TM components reduced cisplatin sensitivity

**Fig. 5:**
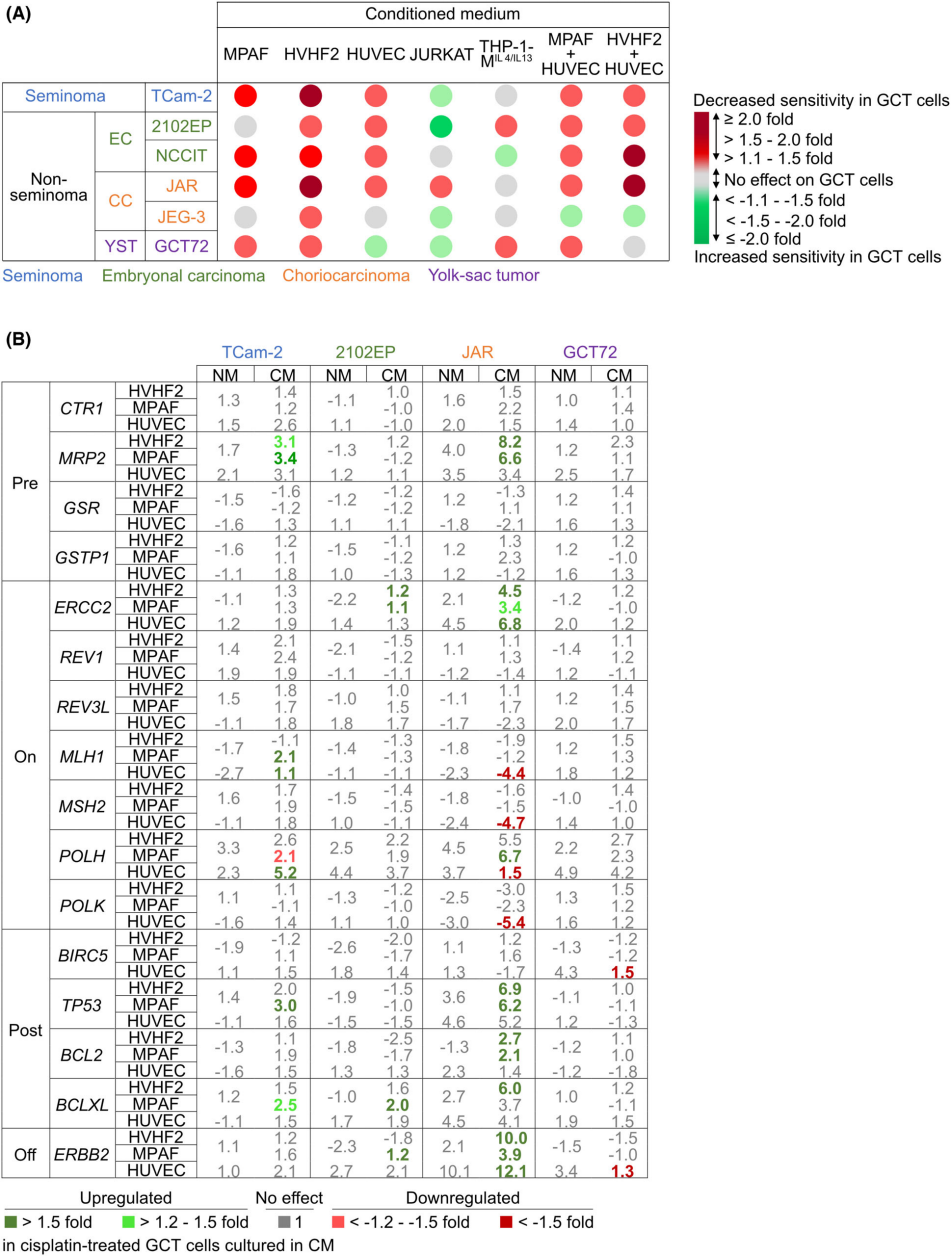
Conditioned medium from fibroblasts and endothelial cells diminishes cisplatin sensitivity. (A) Relative cell viability after 48 h of cisplatin treatment in GCT cells (TCam‐2, 2102EP, NCCIT, JAR, JEG‐3, GCT72) being pretreated for 24 h with conditioned medium from fibroblasts (MPAF and HVHF2), endothelial cells (HUVEC), T‐lymphocytes (JURKAT), M^IL4/IL13^ macrophages (THP‐1‐M^IL4/IL13^) or the combination of fibroblasts and endothelial cells. Indicated as fold change compared with respective cisplatin‐treated condition without pretreatment (*n* = 4). (B) Table indicating relative fold change of genes involved during the development of cisplatin resistance (pretarget: *CTR1*, *MRP2*, *GSR*, *GSTP1*; on‐target: *ERCC2*, *REV1*, *MLH1*, *MSH2*, *POLH*, *POLK*; posttarget: *BIRC5*, *TP53*, *BCL2*, *BCLXL*; off‐target: *ERBB2*) after cisplatin treatment in TCam‐2, 2102EP, JAR, and GCT72 cultivated in standard cultivation medium or conditioned medium from fibroblasts (HVHF2 and MPAF) or endothelial cells (HUVEC) as measured by qRT‐PCR. Differences in fold change between cisplatin‐treated GCT cell lines cultivated in conditioned medium compared with standard cultivation medium indicated in green (> 1.5) and red (< −1.5) (*n* = 3). *GAPDH* and *ACTB* served as housekeeping genes. GCT, germ cell tumor.

Next, we aimed to extrapolate our findings by investigating the effect of microenvironment component‐CM on cisplatin sensitivity of GCT cells. Cell viability assays revealed that from all tested microenvironmental components especially fibroblast‐ and HUVEC‐CM (and both combined) reduced the sensitivity of most GCT cells toward cisplatin (Fig. 5A). Hence, to identify the cause of decreased cisplatin sensitivity being induced in GCT cells due to predisposition to CM, we further evaluated genes known to be involved during the development of cisplatin resistance. As such, several molecular mechanisms of cisplatin resistance have been identified and defined by Galluzzi et al. in various tumor entities during the past four decades, for example, decreased cisplatin uptake combined with increased detoxification (‘pre‐target’), as well as decreased DNA damage response (‘on‐target’), decreased apoptosis induction (‘post‐target’), and circumvention of cisplatin‐induced damage response through compensating pathways (‘off‐target’) [5, 66]. Since cultivation in CM from fibroblasts and endothelial cells resulted in a decreased cisplatin sensitivity in GCT cells, we asked if the expression of postulated cisplatin resistance factors was altered in cisplatin‐treated GCT cell lines (TCam‐2, 2102EP, JAR, and GCT72) cultured in either standard culture medium or CM from fibroblasts or endothelial cells. Cisplatin treatment (24 h, LD_50 48 h_) alone resulted in elevated *MRP2*, *POLH*, *TP53*, *ERBB2* and decreased *GSR* and *MLH1* gene expression in most GCT cell lines (Fig. 5B). Though, comparing cisplatin‐treated GCT cells cultured in CM with cells in standard medium, *MRP2*, *ERCC2*, *TP53*, *BCL2*, *BCLXL*, and *ERBB2* were upregulated in TCam‐2, 2102EP, and JAR cells in fibroblast‐CM. Additionally, cisplatin‐treated JAR cells in HUVEC‐CM downregulated ‘on‐target’ marker genes *MLH1*, *MSH2*, *POLH*, and *POLK*. The tested CM provoked only marginal effects in cisplatin‐treated GCT72 (Fig. 5B). In conclusion, cisplatin treatment under the influence of CM from fibroblasts and endothelial cells led to deregulation of genes involved in ‘post‐’ (*TP53*, *BCL2*, *BCLXL*) and ‘off‐target’ (*ERBB2*) resistance mechanisms in most analyzed GCT cell lines. Additionally, co‐cultivation with fibroblasts and endothelial cells resulted in the highest number of deregulated genes in GCT cells compared to the interaction with immune cells, further demonstrating their strong influence on GCT cells (Fig. S7H).

### 3.4 GCT‐derived conditioned medium polarizes macrophage differentiation

Since factors involved in the ECM organization were also secreted by GCT cell lines, we asked whether the CM from GCT cell lines had an influence on macrophages as a cell type with high plasticity. Macrophages can be classified into M^IFNg/LPS^ or M^IL4/IL13^ macrophages when treated with IFNg or IL4, representing either the inflammation responsive or immune suppressive phenotype, respectively [67]. Thus, in our similarly differentiated macrophages M^IFNg/LPS^ or M^IL4/IL13^_,_ we evaluated changes in gene expression of known macrophage marker genes and verified the successful differentiation of THP‐1 cells into the described states as described by Genin et al. [28] (Fig. S10A,B). Next, we evaluated the effect of GCT‐CM on the differentiation of THP‐1 macrophages (Fig. S10C). Specifically, compared with THP‐1 macrophages, treatment with CM elevated *CD86*, *CXCL10*, *FN1*, *IL10*, *IL12B*, *IL1B*, as well as *MERTK* and decreased *CCL22* expression in at least three of the four tested GCT‐CM (Fig. S10D,E). Here, *FN1* is of specific interest, showing the highest induction of gene expression, thereby implying that GCT cells secrete factors that enhance adhesion of THP‐1 macrophages.

**Fig. 6:**
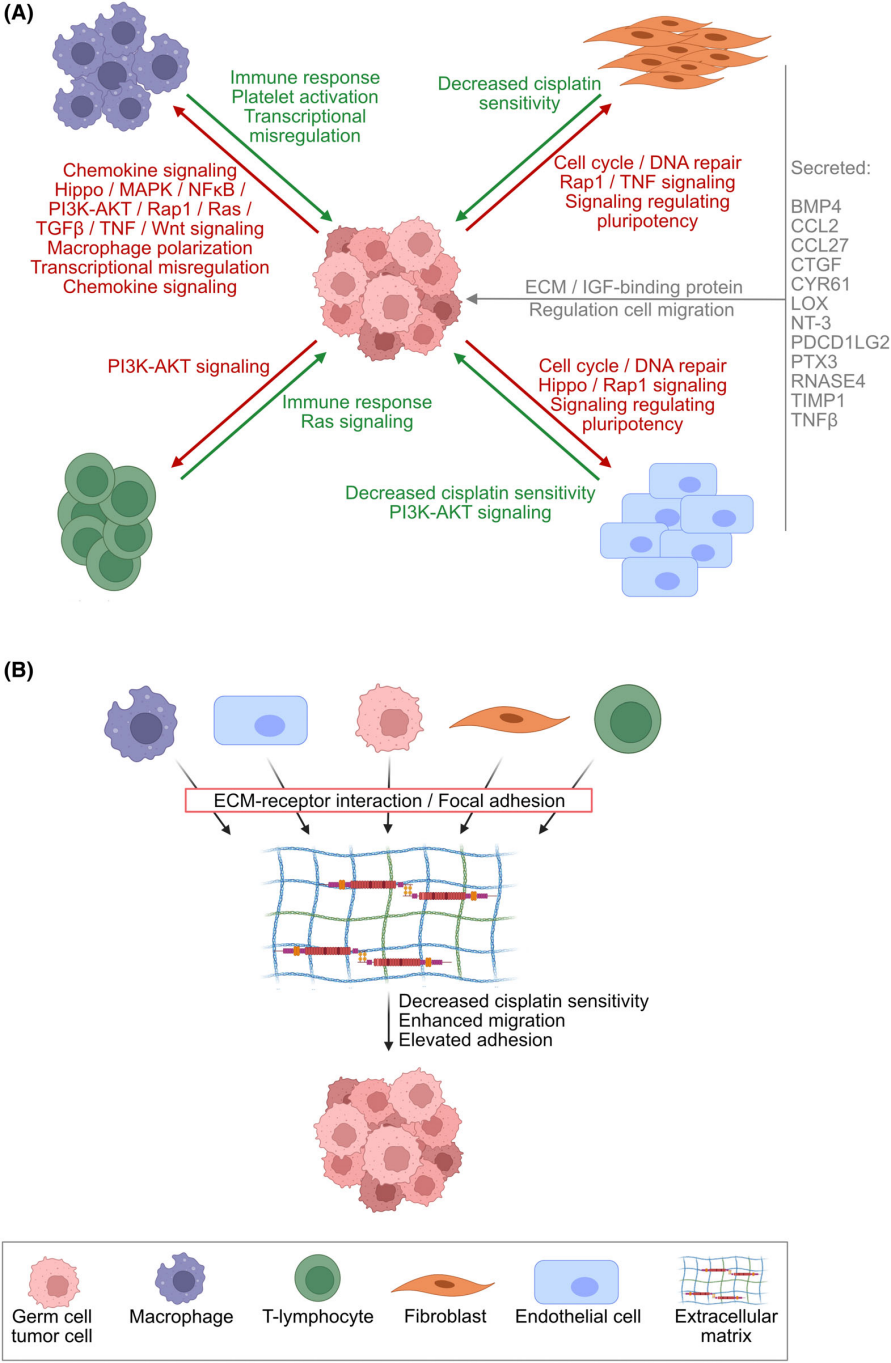
Interaction between GCT cells and microenvironmental components. Graphical illustration of the main findings of this study. Created with BioRender.com. (A) Influence TM components (THP‐1‐M^IL4/IL13^, JURKAT, HUVEC, and HVHF2) on GCT cells (TCam‐2, 2102EP, JAR, and GCT72) and vice versa. (B) Influence of the ECM components collagen I/collagen IV and fibronectin on GCT cells. ECM, extracellular matrix; GCT, germ cell tumor; TM, tumor microenvironment.

Though a tendency toward a polarization into a M^IL4/IL13^‐like macrophage could be noted, the variance of changes in gene expression of related marker genes over time indicates not only the difficulty to distinguish between M^IFNg/LPS^ and M^IL4/IL13^ macrophages, but also points at a phenotypic plasticity of macrophages dependent on time and contextual cues from surrounding tumor as well as TM cells (Fig. 6A). Nevertheless, this experimental approach revealed that GCT cells themselves are able to adjust their neighboring immune cells through the secretion of different cues in a time‐dependent manner. As such, a heterogeneous tumor‐associated macrophage population within a (mixed) GCT could result in a pro‐tumorigenic environment through modulating not only GCT cells, but also surrounding microenvironmental cells, inferentially supporting tumor growth and diminishing sensitivity toward cisplatin‐based chemotherapy.

## 4 Discussion

This study investigated the cross talk between GCT cells with their surrounding TM cells. Based on proteomics and transcriptomics and by utilizing seven GCT cell lines representing seminoma, EC, CC, and YST, as well as five TM cell lines serving as a model for macrophages, T‐lymphocytes, endothelial cells, and fibroblasts, we could identify signaling cascades relevant during the interaction between GCT cells and TM components. Analysis of factors commonly secreted from all GCT cell lines revealed cell surface and signaling molecules being most relevant, while TM components generally secreted factors involved in adherens junction (Fig. 6). Though, investigating each cell line separately accentuates the importance of every individual cell type and its influence on neighboring GCT and TM cells. As such, factors involved in the regulation of immune response and T‐cell activation, as well as leukocyte differentiation were specifically secreted from M^IL4/IL13^ macrophages, while endothelial cells and fibroblasts secreted factors important for integrin binding or IGF receptor signaling pathway, respectively. In line, cytokines secreted by pro‐inflammatory macrophages, such as TNF‐α, IL1, and IL6, have been reported to induce cisplatin resistance and promote metastasis through activating various signaling pathways [68, 69]. Even though CM from THP‐1‐M^IL4/IL13^ did not alter cisplatin sensitivity in GCT cell lines, Genin et al. observed a reduced etoposide‐induced apoptosis rate in the presence of THP‐1‐M^IL4/IL13^ macrophages, while being increased in HepG2 and A549 cancer cells co‐cultured with THP‐1‐M^IFNg/LPS^ macrophages [28].

In this study, the influence of the secretome on the transcriptome of the opposing cell population has been studied. Indeed, a correlation between secreted factors from the GCT or TM cells and resulting changes in corresponding signaling cascades in the opposing cell population could be noted. Specifically, most secreted factors from GCT cells and TM components eventually led to increased gene expression in signaling pathways relevant for cell adhesion, cell migration, or ECM, thus indicating a tumor‐promoting environment (Fig. 6). Klein et al. identified transcripts encoding pro‐inflammatory cytokines (*IL1B*, *IL6*, and *TNF*), anti‐inflammatory cytokines (*TGFB1*), Th1‐driven cytokines (*IL2* and *IFNG*), and chemokines (*CXCL13*, *CXCL10*, and *CXCL5*) to be significantly elevated in GCT and GCNIS tissues compared with hypospermatogenesis, thereby suggesting a pro‐tumorigenic environment. Though all these conditions are known to be infiltrated with T‐cells, these data suggested a pro‐tumorigenic environment [70]. Later, the authors used a co‐culture of TCam‐2 seminoma cells with peripheral blood mononuclear cells (PBMCs) and observed elevated *IL6* expression in TCam‐2 cells after direct contact with PBMCs, pointing at the potential of the seminoma cell line to directly shape its surrounding microenvironment [71]. Of note, regarding potential reprogramming of GCT cells due to microenvironmental cues, we have previously reported that the seminoma cell line TCam‐2 differentiates under TGF‐β1, EGF, and FGF4 application for 8 days directly into a mixed non‐seminoma (no EC component) [72] or could be reprogrammed into an EC‐like cell fate after xenotransplantation into the murine flank for at least 4 weeks [73]. However, this current study could not observe signs of a seminoma‐to‐EC‐transition, since putative driver genes (*NODAL*, *DNMT3B*, *DPPA3*, and *GAL*) were rather downregulated upon co‐culture of TCam‐2 cells with TM components compared with TCam‐2 mono‐cultures. Additionally, since differentiation factors remained mostly unchanged (Table S3Z), the differentiation into a mixed non‐seminoma could be excluded. These findings could be explained by the considerably shorter time point compared with the previously described *in vivo* reprogramming models.

Transcriptome‐wide analyses of co‐cultured GCT and TM cells led us to conclude that genes involved in the ECM organization were upregulated upon co‐culture in both cell populations. The ECM is the major non‐cellular component of the TM and plays a key role in tumor progression and resistance to chemotherapy drugs [74]. Composed of laminin, collagen IV, collagen I, fibronectin, and vitronectin, the ECM in GCT cell lines has been shown to be responsible for tissue integrity and modulated cell proliferation, differentiation, and migration by interaction with integrin and non‐integrin receptors [75]. Hence, to identify the most relevant secreted ECM components, MS‐based secretome data from GCT cells and TM components were re‐analyzed by accentuating lysine and proline‐hydroxylated proteins (Table S3W,X). Indeed, specifically in single proteins, such as collagens and fibronectin, a significant gain in identified peptides has been observed (Table S3W,X). Next, based on the identified ECM components, as well as previously described components, we evaluated the influence of five different ECM components (collagen I, collagen IV, fibronectin, hyaluronic acid, and laminin) on GCT sensitivity to cisplatin, adhesion, and migration. Indeed, cultivation of GCT cells collagen I/IV‐ or fibronectin‐coated culture plates decreased cisplatin sensitivity, while increasing adhesive and migratory capacity of most GCT cell lines (Fig. 6B). Even though we did not observe an influence of laminin coating on neither cisplatin sensitivity, nor migration or adhesion of GCT cells, Andjilani et al. noted enhanced cisplatin‐induced apoptosis in NCCIT cells grown on 8 μg·cm^−2^ laminin‐coated culture plates (compared with 150 μg·cm^−2^ used in the present study) [76]. In conclusion, components of the ECM (collagen I, collagen IV, and fibronectin) favored a pro‐tumorigenic environment by decreasing the cisplatin sensitivity, as well as enhancing the migratory and adhesive capacity, thereby putatively allowing phenotypic plasticity in GCT cells, for example, by promoting the EMT (Fig. 6B). With this regard, co‐culture of MDA‐MB‐231 breast cancer cells with M1 macrophages resulted in a mesenchymal‐to‐epithelial‐transition, while co‐culture with M2‐like macrophages supported an EMT shift in MCF‐7 cells [77]. Though, via imaging mass spectrometry, Chang et al. observed a considerable co‐localization of platinum (^195^Pt) and collagen I in patient‐derived pancreatic cancer xenograft models. Hence, the decreased cisplatin sensitivity in GCT cells cultured on collagen I/IV or fibronectin could partly be explained by a decreased bioactivity of cisplatin, which is bound and immobilized by the ECM components [78].

Our further observations indicated reduced cisplatin sensitivity in GCT cells under the influence of CM from TM components, specifically originating from fibroblasts and endothelial cells. As such, a fibronectin‐rich ECM has been reported to be produced by CAFs upon co‐culture with prostate cancer cells, thereby directing cancer migration [79]. This is in line with our observation that secreted factors from fibroblasts led to increased expression of gene sets in GCT cell lines relevant for cell‐matrix adhesion, collagen fibril organization, or ECM organization/disassembly (Fig. 6 B). Regarding the latter observation and representing a double‐edged sword depending on contextual cues, CAFs have the potential to degrade stroma ECM by releasing MMPs [80, 81], while CAFs from cervical cancer synthesized high amounts of laminin [82].

This study investigated the interaction between microenvironmental components and GCT cell lines; however, what should be studied in the near future is the interaction between the TM cell populations. As such, activated immune cells have been shown to secrete IL‐1β, which in turn resulted in normal dermal fibroblasts to shift into a pro‐inflammatory CAF in an NF‐κB‐dependent manner [83]. To our knowledge, no study has shown the cross talk between the different TM components and the resulting consequence for a tumor‐promoting environment for GCT cells.

According to Hunter et al., ‘microenvironmental’ interactions relate to direct contact of tumor cells with adjacent non‐tumor cells, while ‘macroenvironmental’ interactions include interactions, in which the tumor cell indirectly influences more distant cells [84]. Thus, the identification of the spatial organization of tumor cells within their proximal and distant microenvironment should be a focus of future research activities [84]. Additionally, even though the prognostic value of CAFs and immune cells still needs to be unraveled in GCTs, several therapeutic options targeting immune cells or stromal components [85] should gain more research emphasis for the treatment of (cisplatin‐resistant) GCT patients.

## 5 Conclusion

This study sheds light into the interaction between GCT cells and their circumjacent TM. Cisplatin sensitivity was reduced in GCT cells cultured in CM from fibroblasts and endothelial cells, probably due to the deregulation of genes involved in the induction of apoptosis.

Vice versa, CM from GCT cell lines did influence macrophage polarization through increased expression of genes commonly known to be marker for M^IL4/IL13^ polarization. However, the time kinetics emphasize that the macrophages rather react to their environment in a time‐ and context‐dependent manner than following a defined route of polarization.

Further, we observed that the crosstalk between GCT and TM cells could result in the deposition of the ECM and alter its stiffness, thereby eventually supporting a tumor‐promoting environment. GCT cells cultivated on collagen I / IV and fibronectin precoated plates displayed significantly enhanced migratory and adhesive capacity as well as decreased cisplatin sensitivity. Hence, our observations indicate that targeting the ECM [86] might be a novel therapeutic option combined with cisplatin‐based chemotherapy in GCTs.

## Conflict of interest

The authors declare no conflict of interest.

## Author contributions

MAS contributed to data curation; formal analysis; investigation; methodology; software; validation; visualization; writing—original draft; and writing—review and editing. KE, AS, GFL, GAW contributed to data curation; formal analysis; and investigation. AB, EOK, DD, SA, CW, HH, CM, KS, and KR contributed to methodology. CS contributed to data curation and formal analysis. PP and GP contributed to investigation and methodology. EM contributed to formal analysis; methodology; and writing—review and editing. DN contributed to conceptualization; data curation; formal analysis; methodology; project administration; resources; software; supervision; visualization; and writing—review and editing.

## Supporting information


**Fig. S1.** Identification of secreted factors from GCT and microenvironmental cells.
**Fig. S2.** Enrichment analysis of the GCT secretome.
**Fig. S3.** Enrichment analysis of the microenvironment secretome.
**Fig. S4.** Identification of factors secreted by either GCT or microenvironmental cells.
**Fig. S5.** 3D hanging drop co‐culture of GCT cells with microenvironmental cells results in morphological changes.
**Fig. S6.** Separation of 3D co‐cultured GCT cells and TM components by flow cytometry.
**Fig. S7.** Analyses of transcriptome‐wide changes upon 3D co‐culture of GCT and microenvironmental cell lines.
**Fig. S8.** Interaction analysis of commonly deregulated genes in microenvironmental cells upon 3D co‐culture with GCT cell lines.
**Fig. S9.** Proteins were identified from conditioned medium via mass‐spectrometric analysis.
**Fig. S10.** Secreted factors from GCT cell lines influence macrophage differentiation.

**Table S1.** Studied cell lines including appropriate culture conditions.

**Table S2.** Sequences of used oligonucleotides.

**Table S3.** Analyzed secretome and transcriptome data to evaluate the cross talk between GCT cells and microenvironmental components.

**Data S1.** Supporting information.

## Data Availability

The datasets supporting the conclusions of this article are available in the NCBI Gene Expression Omnibus (GEO) database repository [GSE195794; https://www.ncbi.nlm.nih.gov/geo/] and the ProteomeXchange database [PDX031329; http://proteomecentral.proteomexchange.org/cgi/GetDataset].
