# Supplementary material for: Profiling the 3D interaction between germ cell tumors and microenvironmental cells at the transcriptome and secretome level
Source: Mol Oncol. 2022 Jul 26;16(17):3107–27. doi: 10.1002/1878-0261.13282 (PMC9441004; doi:10.1002/1878-0261.13282)
Supplement: Supplementary file 2 — Table S1. Studied cell lines including appropriate culture conditions. [file MOL2-16-3107-s003.pdf]

Cell line	Species	Entity	Medium	Supplements	Kindly provided by
1411H	Human	Embryonal carcinoma / Yolk-sac tumor	DMEM (1x) + GlutaMAX-I	10 % FBS, 1 % P/S (10,000 U), 1 % L-Glutamin (200 mM)	Dr. Matthew Murray (University of Cambridge, Cambridge, United Kingdom)
2102EP	Human	Embryonal carcinoma	DMEM (1x) + GlutaMAX-I	10 % FBS, 1 % P/S (10,000 U), 1 % L-Glutamin (200 mM)	Dr. Christoph Oing (University Hospital Eppendorf, Hamburg, Germany)
GCT72	Human	Yolk-sac tumor	RPMI Medium 1640 (1x)	10 % FBS, 1 % P/S (10,000 U), 1 % L-Glutamin (200 mM)	Dr. Thomas Müller (University Clinic for Internal Medicine IV, Hematology/Oncology, Medical Faculty of Martin Luther University Halle-Wittenberg, Halle (Saale), Germany)
HUVEC	Human	Endothelial cells	Endothelial Cell Growth Medium 2	Manufacturers provided supplements	Prof. Dr. Gerhard Fritz (Institute of Toxicology, Medical Faculty, Heinrich Heine University Duesseldorf, Düsseldorf, Germany)
HVHF2	Human	Fibroblasts	DMEM (1x) + GlutaMAX-I	10 % FBS, 1 % P/S (10,000 U), 1 % L-Glutamin (200 mM), 1% non-ess. aa (100x)	Clinic for dermatology, University Hospital Düsseldorf, Germany
JAR	Human	Choriocarcinoma	DMEM (1x) + GlutaMAX-I	10 % FBS, 1 % P/S (10,000 U), 1 % L-Glutamin (200 mM)	ATCC, #HTB-144
JEG-3	Human	Choriocarcinoma	DMEM (1x) + GlutaMAX-I	10 % FBS, 1 % P/S (10,000 U), 1 % L-Glutamin (200 mM)	ATCC, #HTB-36
JURKAT	Human	T lymphocytes	RPMI Medium 1640 (1x)	10 % FBS, 1 % P/S (10,000 U), 1 % L-Glutamin (200 mM)	Prof. Dr. Rüdiger Sorg (Institute for Transplantation Diagnostics and Cell Therapeutics, Faculty of Medicine, Heinrich-Heine-University, Düsseldorf, Germany)
MPAF	Human	Fibroblasts	DMEM (1x) + GlutaMAX-I	10 % FBS, 1 % P/S (10,000 U), 1 % L-Glutamin (200 mM), 1% non-ess. aa (100x)	Dr. Michael Peitz (Life & Brain, Department of Reconstructive Neurobiology, Bonn, Germany)
NCCIT	Human	Embryonal carcinoma	RPMI Medium 1640 (1x)	10 % FBS, 1 % P/S (10,000 U), 1 % L-Glutamin (200 mM)	Dr. Christoph Oing (University Hospital Eppendorf, Hamburg, Germany)
TCam-2	Human	Seminoma	RPMI Medium 1640 (1x)	10 % FBS, 1 % P/S (10,000 U), 1 % L-Glutamin (200 mM)	Dr. Janet Shipley (Institute of Cancer Research, Sutton, England)
THP-1	Human	Monocytes	RPMI Medium 1640 (1x)	10 % FBS, 1 % P/S (10,000 U), 1 % L-Glutamin (200 mM)	Merck, #88081201
