# Supplementary material for: Profiling the 3D interaction between germ cell tumors and microenvironmental cells at the transcriptome and secretome level
Source: Mol Oncol. 2022 Jul 26;16(17):3107–27. doi: 10.1002/1878-0261.13282 (PMC9441004; doi:10.1002/1878-0261.13282)
Supplement: Supplementary file 3 — Table S2. Sequences of used oligonucleotides. [file MOL2-16-3107-s005.pdf]

| Gene | Forward primer | Reverse primer | Tan | Cycles |
|---|---|---|---|---|
| | | | | |
| BCL2 | CCTGTGGATGACTGAGTACCTG | CAGAGGCCGCATGCTGGG | 60°C | 45 |
| BCLXL | TAAACTGGGGTCGCATTGTG | AGGTAAGTGGCCATCCAAGC | 60°C | 45 |
| BIRC5 | AGGACCACCGCATCTCTACAT | AAGTCTGGCTCGTTCTCAGTG | 60°C | 45 |
| CCL1 | TGCAGGTACCCTTCTCCAGA | TGCCTCTGAACCCATCCAAC | 60°C | 45 |
| CCL17 | CACTGAAGATGCTGGCCCTG | TCTGGTACCACGTCTTCAGC | 60°C | 45 |
| CCL22 | ATCGCCTACAGACTGCACTC | GACGGTAACGGACGTAATCAC | 60°C | 45 |
| CD14 | ACGCCAGAACCTTGTGAGC | GCATGGATCTCCACCTCTACTG | 60°C | 45 |
| CD163 | TTTGTCAACTTGAGTCCCTTCAC | TCCCGCTACACTTGTTTTCAC | 60°C | 45 |
| CD206 | CTACAAGGGATCGGGTTTATGGA | TTGGCATTGCCTAGTAGCGTA | 60°C | 45 |
| CD36 | CTTTGGCTTAATGAGACTGGGAC | GCAACAAACATCACCACACCA | 60°C | 45 |
| CD44 | TGCCGCTTTGCAGGTGTAT | GGCCTCCGTCCGAGAGA | 60°C | 45 |
| CD6 | CCGGCAGGATGTACTACTCAT | GGACAGATTGTGCAAACTCCG | 60°C | 45 |
| CD80 | GGGCACATACGAGTGTGTTGT | TCAGCTTTGACTGATAACGTCAC | 60°C | 45 |
| CD86 | CTGCTCATCTATACACGGTTACC | GGAAACGTCGTACAGTTCTGTG | 60°C | 45 |
| CTR1 | GGGGATGAGCTATATGGACTCC | TCACCAAACCGGAAAACAGTAG | 60°C | 45 |
| CXCL10 | TTCAAGGAGTACCTCTCTCTAG | CTGGATTCAGACATCTCTTCTC | 60°C | 45 |
| DCN | ATGAAGGCCACTATCATCCTCC | GTCGCGGTCATCAGGAACTT | 60°C | 45 |
| EpCAM/CD326 | GCAGCTCAGGAAGAATGTG | CAGCCAGCTTTGAGCAAATGAC | 60°C | 45 |
| ERBB2 | CCAGCTGGCTCTCACACTG | AGCCCTTACACATCGGAGAAC | 60°C | 45 |
| ERCC2 | GTCGATGGGAAATGCCACAG | GTCATCCAGGTTGTAGATGCC | 60°C | 45 |
| FN1 | CGGTGGCTGTCAGTCAAAG | AAACCTCGGCTTCCTCCATAA | 60°C | 45 |
| GAPDH | TGCCAAATATGATGACATCAAGAA | GGAGTGGGTGTCGCTGTTG | 60°C | 45 |
| GSR | TTCCAGAATACCAACGTCAAAGG | GTTTTCGGCCAGCAGCTATTG | 60°C | 45 |
| GSTP1 | CCCTACACCGTGGTCTATTTCC | CAGGAGGCTTTGAGTGAGC | 60°C | 45 |
| IL10 | GACTTTAAGGGTTACCTGGGTTG | TCACATGCGCCTTGATGTCTG | 60°C | 45 |
| IL12B | GCGAGGTTCTAAGCCATTCG | TGATTGTCGTCAGCCACCAG | 60°C | 45 |
| IL1b | AGCTACGAATCTCCGACCAC | CGTTATCCCATGTGTCGAAGAA | 60°C | 45 |
| IL6 | ACTCACCTCTTCAGAACGAATTG | CCATCTTTGGAAGGTTCAGGTTG | 60°C | 45 |
| MERTK | GTGCAGCGTTCAGACAATGG | TGACAGGTGAGGTTGAAGGC | 60°C | 45 |
| MHL1 | CTCTTCATCAACCATCGTCTGG | GCAAATAGGCTGCATACACTGTT | 60°C | 45 |
| MRP2 | CCCTGCTGTTCGATATACCAATC | TCGAGAGAATCCAGAATAGGGAC | 60°C | 45 |
| MSH2 | AGGCATCCAAGGAGAATGATTG | GGAATCCACATACCCAACTCCAA | 60°C | 45 |
| PECAM-1/CD31 | ACCGTGACGGAATCCTTCTCT | GCTGGACTCCACTTTGCAC | 60°C | 45 |
| POLH | CTGGCACAAGTTCGTGAGTC | GCAACAAGTCTGCCGAGATAG | 60°C | 45 |
| POLK | GCCATGCCAGGATTTATTGCT | GCTTCATCAAGACTCATGGCC | 60°C | 45 |
| REV1 | GATGGAGGAAGCGAGCTGAAA | CCTTCTGCATAGCAGCATCTG | 60°C | 45 |
| REV3L | GTGGATGCTGTAGCTGCTGAT | ATGGCCTGTAGACCAGGGTTT | 60°C | 45 |
| TGFB1 | ACCCGTGTTGCTCTCCCG | CAACCACTGCCGCACAAC | 60°C | 45 |
| TGFB2 | AGGGATCTAGGGTGGAAATGGA | GCAGCAAGGAGAAGCAGATG | 60°C | 45 |
| TGFB3 | ATGTCACACCTTTCAGCCCA | AGATGAGGGTTGTGGTGATCC | 60°C | 45 |
| TNFSF14 | CGAAGGTCTCACGAGGTCAA | AGTAGTAGCCAGCTTTGGTG | 60°C | 45 |
| TP53 | CAGCACATGACGGAGGTTGT | TCATCCAAATACTCCACACGC | 60°C | 45 |
