# Supplementary material for: Profiling the 3D interaction between germ cell tumors and microenvironmental cells at the transcriptome and secretome level
Source: Mol Oncol. 2022 Jul 26;16(17):3107–27. doi: 10.1002/1878-0261.13282 (PMC9441004; doi:10.1002/1878-0261.13282)
Supplement: Supplementary file 5 — Data S1. Supporting information. [file MOL2-16-3107-s001.docx]

**Supporting information**

Fig. S1: Identification of secreted factors from GCT and microenvironmental cells. A) Validation of THP-1-M^IL4/IL13^ polarization by measuring A) *CD14*, *CD36*, *CD163*, *CD206*, and *FN1* expression by qRT-PCR (n = 6) or B) CD36, CD14, or CD68 protein levels by antibody-staining with subsequent flow cytometry compared to undifferentiated THP-1 cells (n = 2). *GAPDH* served as a housekeeping gene. C) Microscopic brightfield pictures of GCT cell lines (seminoma: TCam-2; embryonal carcinoma: 2102EP, NCCIT; choriocarcinoma: JAR, JEG-3; yolk-sac tumor: GCT72; intermediate between EC and YST: 1411H) and TM cells (M^IL4/IL13^ macrophages: THP-1-M^IL4/IL13^; T-lymphocytes: JURKAT; endothelial cells: HUVEC; fibroblasts: HVHF2, MPAF) before supernatants and cell lysates were harvested for further mass spectrometric analyses (n = 3). D) Protein extraction quality control of secreted proteins as indicated by silver staining of each cell line in triplicates. E) Hierarchical cluster analysis (without missing values, Spearman correlation) of secreted proteins found in seven GCT and five TM cell lines as measured by mass spectrometry. 3D Principal component analysis (PCA) visualizing mass spectrometry data (n = 3) from F) GCT cells (TCam-2, 2102EP, NCCIT, GCT72, 1411H, JAR, JEG-3) and G) TM cells (HUVEC, JURKAT, THP-1-M^IL4/IL13^, HVHF2, MPAF). H) Correlogram (Pearson type) representing correlation coefficients between mass spectrometry data of GCT and TM cells. Colored circles indicate the correlation coefficient (R) with positive correlations being displayed in blue and negative correlations in red. Color intensity and the size of the circle are proportional to the correlation coefficients. GCT: germ cell tumor, TM: tumor microenvironment.

Fig. S2: Enrichment analysis of the GCT secretome. STRING-based protein interaction prediction analyses of secreted factors from A) seminoma (TCam-2), B) embryonal carcinoma (2102EP, NCCIT), C) choriocarcinoma (JAR, JEG-3), and D) yolk-sac tumor (GCT72) as measured by mass spectrometry (n = 3).

Fig. S3: Enrichment analysis of the microenvironment secretome. STRING-based protein interaction prediction analyses of secreted factors from A) M^IL4/IL13^ macrophages (THP-1-M^IL4/IL13^), B) endothelial cells (HUVEC), C) T-lymphocytes (JURKAT), and D) fibroblasts (HVHF2, MPAF) as measured by mass spectrometry (n = 3).

Fig. S4: Identification of factors secreted by either GCT or microenvironmental cells. Human cytokine array raw and processed exemplary data. Supernatants from GCT cell lines (TCam-2, 2102EP, JAR, 1411H) and TM cells (THP-1-M^IL4/IL13^, JURKAT, HUVEC, MPAF) were analyzed in quadruplicates. Corresponding membrane layout of both membranes is given in the lower panel. Most prominent dots (marked in red) were used for quantification using ImageJ. GCT: germ cell tumor.

Fig. S5: 3D hanging drop co-culture of GCT cells with microenvironmental cells results in morphological changes. A) Microscopic brightfield pictures of cell aggregates developed from 3D hanging drop mono- or co-cultured GCT- (TCam-2, 2102EP, NCCIT, JAR, JEG-3, GCT72) and/or TM cell lines (HUVEC, JURKAT, THP-1-M^IL4/IL13^, HVHF2, MPAF) after 72 h. Scale bar = 100 µm. B) Quantification of the mono-cultured cell aggregates observed in A) (n = 6). C) 3D hanging-drop co-culture to visualize the interaction between GCT cells and their microenvironment. Confocal microscopy of 3D hanging drop co-cultures of GFP-transduced GCT cells (TCam-2, 2102EP, NCCIT, GCT72, 1411H, JAR, JEG-3) cells with DeepRed-stained fibroblasts (MPAF, HVHF2) or mCherry-transduced endothelial cells (HUVEC), T-lymphocytes (JURKAT), or M^IL4/IL13^ macrophages after 72 h. DAPI counterstaining was used to stain nuclei. Scale bar = 100 µm. GCT: germ cell tumor.

Fig. S6: Separation of 3D co-cultured GCT cells and TM components by flow cytometry. A) Representative gating strategy to sort either GFP-expressing GCT cells or mCherry-expressing endothelial or immune cells for further culture. Validation of successful DeepRed-staining of fibroblasts. B) Representative gating strategy of sorted GFP-expressing GCT cells and mCherry-expressing or DeepRed-stained TM components after 72 h incubation in the 3D hanging drop. Cellular debris and doublets were excluded by using the side-scatter signals (SSC-width and SSC-height) of both cell populations. GCT: germ cell tumor, TM: tumor microenvironment.

Fig. S7: Analyses of transcriptome-wide changes upon 3D co-culture of GCT and microenvironmental cell lines. A) qRT-PCR of the expression of *CD326/ EpCAM* (GCT marker), *CD36* (macrophage marker), *CD44* (marker for TM cells), *CD31/ PECAM1* (endothelial cell marker), *DCN* (fibroblast marker), and *CD6* (T-lymphocyte marker) in TCam-2, 2102EP, NCCIT, JAR, JEG-3, 1411H, GCT72, HUVEC, JURKAT, THP-1-M^IL4/IL13^, HVHF2, and MPAF (n = 3). *GAPDH* served as a housekeeping gene. B) qRT-PCR validation of *CD326/ EpCAM* and *CD44* expression in flow cytometry-sorted cell populations after 72 h 3D co-culture (n = 3). *GAPDH* served as a housekeeping gene. C) 3D PCA and D) hierarchical cluster analysis (Spearman correlation) visualizing RNA-seq data from sorted GCT cells (TCam-2, 2102EP, JAR, GCT72) after co-culture with TM cells (HUVEC, JURKAT, THP-1-M^IL4/IL13^, HVHF2) for 72 h in comparison with their respective 3D monoculture. E) 3D PCA and (F) hierarchical cluster analysis (Spearman correlation) visualizing RNA-sequencing data from sorted TM cells (HUVEC, JURKAT, THP-1-M^IL4/IL13^, HVHF2) after co-culture with GCT cells (TCam-2, 2102EP, JAR, GCT72) for 72 h in comparison with their respective 3D monoculture. Volcano plots indicating the absolute number of up- (green) and downregulated (red) genes (> log_2_ 1.5 fold change, p-value FDR < 0.05) in G) GCT cell lines co-cultured with TM cells, and H) vice versa. GCT: germ cell tumor, TM: tumor microenvironment, PCA: principal component analyses, FDR: False discovery rate.

Fig. S8: Interaction analysis of commonly deregulated genes in microenvironmental cells upon 3D co-culture with GCT cell lines. STRING-based protein interaction prediction analyses of upregulated genes in A) THP-1-M^IL4/IL13^, B) HVHF2, C) HUVEC, and D) JURKAT cells co-cultured with GCT cell lines for 72 h. GCT: germ cell tumor.

Fig. S9: Proteins were identified from conditioned medium via mass-spectrometric analysis. A) Protein intensities of proteins predicted to be secreted for each cell line were summed up and the intensity proportion of hydroxylated and potential extracellular matrix (ECM) proteins calculated for each cell line (n = 3). B) The intensity proportion of the top 150 abundant proteins predicted to be secreted was visualized by voronoi treemaps (n = 3). Treemaps were built upon information of extracellular matrix / extracellular vesicle annotation and lysine and proline hydroxylation detected by mass spectrometry.

Fig. S10: Secreted factors from GCT cell lines influence macrophage differentiation. A) Graphical illustration of the differentiation protocol from THP-1 monocytes to macrophages and subsequent polarization into M^IFNg/LPS^ or M^IL4/IL13^ macrophages (created with BioRender.com) including raw exemplary microscopic brightfield pictures. B) Relative gene expression of *CCL1*, *CCL17*, *CCL22*, *CD80*, *CD86*, *CD163*, *CD206*, *CXCL10*, *FN1*, *IL10*, *IL12B*, *IL1B*, *MERTK*, *TGFB1*, *TGFB2*, *TGFB3*, and *TNFSF14* in THP-1, THP-1-M^IFNg/LPS^, and THP-1-M^IL4/IL13^ as measured by qRT-PCR (n = 3). *GAPDH* and *ACTB* served as housekeeping genes. C) Graphical illustration of THP-1-M0 cells treated with conditioned medium from GCT cells TCam-2, 2102EP, JAR, or GCT72 for up to 72 h (created with BioRender.com) including raw exemplary microscopic brightfield pictures. D) Relative gene expression of marker genes identified in B) in THP-1-M0 cells treated with conditioned medium from GCT cell lines for 24 h, 48 h, and 72 h as measured by qRT-PCR and indicated as fold change compared to THP-1-M0 (n = 3). *GAPDH* and *ACTB* served as housekeeping genes. Differences in fold change between THP-1-M0 treated with conditioned medium from GCT cell lines compared to THP-1-M0 indicated in green (> 1.5; 72 h) and red (< -1.5; 72 h). E) Summarizing table indicating commonly deregulated genes found in D) in at least three of the four used conditioned media, in conditioned media from seminoma and EC, or in conditioned media from extraembryonic GCT cell lines (YST, CC). GCT: germ cell tumor, YST: yolk-sac tumor, CC: choriocarcinoma.

Table S1: Studied cell lines including appropriate culture conditions.

Table S2: Sequences of used oligonucleotides.

~~Data S1~~ Table S3: Analyzed secretome and transcriptome data to evaluate the cross-talk between GCT cells and microenvironmental components. A - L: Raw data of mass spectrometry analysis of cell lysates and secretomes isolated from seven GCT cell lines (TCam-2, 2102EP; JAR, JEG-3, GCT72, 1411H) and five TM cell lines (THP-1-M^IL4/IL13^, JURKAT, HUVEC, HVHF2, MPAF) (n = 3). M - V: Processed data from mass spectrometry and cytokine arrays indicating secreted factors from GCT cell lines (TCam-2, 2102EP, NCCIT, JAR, JEG-3, 1411H, GCT72) and TM cell lines (THP-1-M^IL4/IL13^, JURKAT, HUVEC, HVHF2, MPAF) (n = 3). W, X: Depicted transcriptome data (log_2_ fold change compared to mono-culture) regarding the expression of putative driver genes of reprogramming or differentiation factors and integrins. Y, Z: Raw data of mass spectrometry analyses of secretomes isolated from seven GCT cell lines (TCam-2, 2102EP; JAR, JEG-3, GCT72, 1411H) and five TM cell lines (THP-1-M^IL4/IL13^, JURKAT, HUVEC, HVHF2, MPAF) with the emphasis on lysine and proline hydroxylated proteins and peptides (n = 3). GCT: germ cell tumor, TM: tumor microenvironment.
